# Monitoring urban biological invasions using citizen science: the polyphagous shot hole borer (*Euwallacea fornicatus*)

**DOI:** 10.1007/s10340-024-01744-7

**Published:** 2024-01-27

**Authors:** Luke J. Potgieter, Marc W. Cadotte, Francois Roets, David M. Richardson

**Affiliations:** 1 Centre for Invasion Biology, Department of Botany and Zoology, Stellenbosch University, Stellenbosch, South Africa; 2 Department of Biological Sciences, University of Toronto-Scarborough, 1265 Military Trail, Toronto, ON M1C 1A4 Canada; 3 Department of Conservation Ecology and Entomology, Stellenbosch University, Stellenbosch, South Africa; 4 Department of Invasion Ecology, Institute of Botany, Czech Academy of Sciences, Průhonice, Czech Republic

**Keywords:** Biological invasions, Citizen science, Pests and pathogens, Polyphagous shot hole borer, Monitoring, Urban

## Abstract

Benefits provided by urban trees are increasingly threatened by non-native pests and pathogens. Monitoring of these invasions is critical for the effective management and conservation of urban tree populations. However, a shortage of professionally collected species occurrence data is a major impediment to assessments of biological invasions in urban areas. We applied data from iNaturalist to develop a protocol for monitoring urban biological invasions using the polyphagous shot hole borer (PSHB) invasion in two urban areas of South Africa. iNaturalist records for all known PSHB reproductive host species were used together with data on localities of sites for processing plant biomass to map priority monitoring areas for detecting new and expanding PSHB infestations. Priority monitoring areas were also identified using the distribution of *Acer negundo*, a highly susceptible host that serves as a sentinel species for the detection of PSHB infestations. iNaturalist data provided close to 9000 observations for hosts in which PSHB is known to reproduce in our study area (349 of which were *A. negundo*). High-priority areas for PSHB monitoring include those with the highest density of PSHB reproductive hosts found close to the 140 plant biomass sites identified. We also identified high-priority roads for visual and baited trap surveys, providing operational guidance for practitioners. The monitoring protocol developed in this study highlights the value of citizen or community science data in informing the management of urban biological invasions. It also advocates for the use of platforms such as iNaturalist as essential tools for conservation monitoring in urban landscapes.

**Supplementary Information:**

The online version contains supplementary material available at 10.1007/s10340-024-01744-7.

## Introduction

Biological invasions are one of the five main threats to biodiversity worldwide (IPBES 2019); they can cause widespread ecological and economic damage, negatively impacting the well-being of people. With more than 57% of the global human population living in urban areas, urbanization is a major driver of global environmental change (United Nations 2018). Cities are well-connected transportation hubs that facilitate species dispersal from one region to another, allowing non-native species to spread across the globe. As a result, urban areas receive more non-native species introductions compared to rural or natural areas (Rebele 1994). With the growing urbanization and global interconnectivity of the world's human population, this rise in the introduction of non-native species will continue to increase (Perrings et al. 2010). Anthropogenic activities such as resource supplementation, increased disturbance, and transportation networks provide many opportunities for non-native species to establish and spread. Indeed, many non-native species exhibit strong adaptability to urban environments, enabling them to invade both urban ecosystems and the natural surroundings of towns and cities (Cadotte et al. 2017; Potgieter and Cadotte 2020). As urbanization expands globally, the significance of invasive species' impact on urban environments is increasing, underscoring the urgency for implementing effective strategies to mitigate their effects.

Urban forests are an important component of cities, providing a range of ecological, economic, and social benefits. They play a vital role in maintaining local biodiversity, regulating the urban microclimate, mitigating the urban heat island effect, enhancing air and water quality, and offering aesthetic and recreational opportunities for urban dwellers (Dwyer et al. 1992; Tyrväinen et al. 2005; Willis and Petrokofsky 2017). However, urban forests are also vulnerable to threats from introduced pests and pathogens, which can have significant negative impacts on biodiversity, ecosystem services, and human well-being (Paap et al. 2017). For example, the invasion of emerald ash borer (*Agrilus planipennis*) in the eastern United States and Canada has led to mass mortality of urban trees, negatively affecting human health and property values, while resulting in significant financial costs for treating, removing, and replacing high-value trees (Donovan et al. 2013; Schrader et al. 2021). Urban trees often face higher levels of stress than their rural counterparts, including pollution, soil compaction, and limited water and nutrient availability (Pautasso et al. 2015). These stresses can make urban trees more susceptible to pests and pathogens (Raupp et al. 2006). In addition, the proximity of urban trees to one another, coupled with planting regimes that creates monospecific stands, can facilitate the spread of pests and pathogens, allowing diseases to move quickly through tree populations and cause widespread damage. Indeed, pests and pathogens are becoming increasingly prevalent in urban areas where they are causing significant economic, environmental, and social impacts (Raum et al. 2023).

Limited resources allocated for monitoring non-native species and the increasing exposure of sites to non-native species’ introductions highlight the need for effective protocols for identifying sites most vulnerable to their establishment (Epanchin-Niell et al. 2014). The movement of plant biomass, internationally and within regions, is an important pathway for the spread of pests and pathogens (Meurisse et al. 2019). Sites where plant biomass is handled, processed, and/or disposed of (hereafter referred to as plant biomass sites) are at high risk for pest/pathogen introductions and should be prioritised for monitoring. For example, the transport of nursery stock between nurseries and their customers can facilitate the long-distance transmission of pathogens, serving as a vector for their spread (Liebhold et al. 2012; Ghelardini et al. 2017; Simamora et al. 2018; Puertolas et al. 2021). The disposal of infested plant biomass at formal (and informal) waste facilities without following appropriate handling and phytosanitary measures also presents a major dispersal pathway for pests and pathogens; waste landfills can harbour high species richness and abundance of non-native beetle species (Auclair et al. 2005; Rassati et al. 2015). The transportation of firewood by individuals for recreational activities, cooking, or residential heating purposes can also serve as a pathway for the dispersal of wood-inhabiting pests (Solano et al. 2021). These sites and activities, and consequently vectors and pathways of non-native species spread, are ubiquitous in urban landscapes (Padayachee et al. 2017) and must be well understood and incorporated in strategies for monitoring and surveillance for early detection of pests and pathogens.

Monitoring for pest and pathogen invasions is critical for the effective management and conservation of urban tree populations. Monitoring involves regular surveillance and assessment of the abundance, distribution, and impact of invasive species in urban environments. Data collected through monitoring efforts must inform management decisions and be used to prioritize actions aimed at controlling or eradicating invasive species (Potgieter et al. 2021). However, monitoring the urban forest is challenging. For example, many trees are located on private property and, in some countries, landowners can bar access to authorities attempting to gather monitoring data (Bertolino et al. 2021). Monitoring urban trees also requires time, expertise, and resources, which can be in short supply for local governments and organizations (Roman et al. 2013). However, technological advances like remote sensing and environmental DNA (eDNA) and increasingly popular citizen science initiatives (such as eBird and iNaturalist) are revolutionizing our ability to detect and respond to invasions (Allan et al. 2018). Indeed, citizen science initiatives that promote public participation in large-scale, cost-effective biodiversity identification and monitoring can overcome some of these challenges and serve as key resources for the early detection of pest/pathogen infestations.

Conventional, professional survey methods often fail to adequately assess urban landscapes, resulting in a scarcity of species occurrence data (Ballard et al. 2017) which can hinder urban biological invasion assessments. However, the utilization of citizen science platforms has significantly bolstered the quantity and accessibility of species occurrence data from urban areas (Spear et al. 2017). Consequently, there are currently more data on urban species occurrences than ever before (Silvertown 2009). Indeed, some of these citizen science data have made valuable contributions to the management of biological invasions in urban areas (e.g. Crall et al. 2010). The collection of citizen science data not only addresses the challenges of accessing private land (through actively participating landowners also willing to grant access to other citizen scientists) but can also be easily expanded to encompass multiple cities (Spear et al. 2017).

Globally, iNaturalist stands as one of the most widely embraced citizen science platforms for biodiversity, with more than 3.6 million observers having contributed over 180 million observations (https://www.inaturalist.org; accessed 19 December 2023). Observers can upload geolocated records of any species, and the community aids in validating species identifications. iNaturalist has already made substantial contributions to ecological research, e.g. tracking range expansions of non-native species (Agarwal 2017), identifying new and emerging invasive species (Hiller and Haelewaters 2019), and conducting regular surveillance of invasive species (Larson et al. 2020). Nevertheless, the potential of platforms such as iNaturalist for monitoring invasive species in urban environments has not been extensively investigated.

In this study, we use iNaturalist data to develop a monitoring protocol for the early detection and surveillance of urban biological invasions using the polyphagous shot hole borer (PSHB) in two urban centres of South Africa as a case study. iNaturalist records for PSHB-reproductive host tree species were used together with data on plant biomass sites to develop a monitoring protocol for detecting new and expanding PSHB infestations.

## Methods

### Study taxa

Wood borers are among the most damaging pests worldwide and many species have become successful invaders, often causing substantial economic, ecological, and social costs (Raum et al. 2023). PSHB, *Euwallacea fornicatus* (Coleoptera: Curculionidae: Scolytinae), is an ambrosia beetle native to Southeast Asia (Stouthamer et al. 2017) that has been unintentionally introduced to many parts of the world, including Australia, California, Hawaii, Israel, South Africa, and many European countries, with varying levels of success in containing or eradicating it (van Rooyen et al. 2021). PSHB has a symbiotic relationship with at least three fungal species: *Fusarium euwallaceae*, *Graphium euwallaceae* and *Paracremonium pembeum* (Lynch et al. 2016), which serve as a food source for the adults and their larvae (Freeman et al. 2013). In susceptible hosts, this leads to *Fusarium* Dieback, a disease that, in conjunction with the boring activities of the beetle, can cause branch dieback and tree death (Freeman et al. 2016).

PSHB can establish in a wide range of native and non-native tree species in urban, agricultural, and natural landscapes (van Rooyen et al. 2021). Susceptible tree species can serve as reproductive or non-reproductive hosts for the beetle, showing different responses to infestations. Reproductive host trees are those in which both the beetles and the fungus establish, and where the beetles can reproduce. Susceptible reproductive hosts may eventually be killed by the combined impacts of the beetle and the fungus. Non-reproductive host trees are targeted and attacked by the beetle, leading to the establishment of the fungus, but the beetle does not reproduce; in such trees, the fungus may or may not cause disease but is unlikely to kill the tree (Freeman et al. 2013, 2016; Lynch et al. 2016).

Female beetles are dark brown to black and 1.8–2.5 mm long and winged. Male beetles are reddish-brown, smaller than females at 1.5 mm long, and apterous. The female beetle tunnels into the host tree forming galleries in which eggs are laid. Females lay an average of 32 eggs over their lifetime and the first adults that develop from these can appear after only 22 days under ideal conditions (Cooperband et al. 2016). Females exhibit slower development rates compared to males, although they are produced in significantly larger quantities (Cooperband et al. 2016; Umeda and Paine 2019). Generally, females are fertilized by their brothers inside the galleries after which they emerge through the original entrance tunnel and take flight to seek out new hosts. An unmated female can, however, still establish a gallery and lay eggs. These unfertilised eggs all hatch as haploid males. The female can mate with any of these, and then go on to lay diploid female eggs again (Cooperband et al. 2016). This means that a single unmated female can establish a new colony.

The beetles have special structures at the base of their mandibles called mycangia which are used to transport the spores of their fungal symbionts (Freeman et al. 2013). During the process of establishing brood galleries, adult female beetles burrow into trees, introducing fungal spores that colonize the walls of the galleries. These spores then serve as a nourishing food source for both developing larvae and adult beetles. By infiltrating the vascular tissues of trees, *F. euwallaceae* disrupts the transportation of water and nutrients, leading to branch dieback and tree death, known as *Fusarium* dieback (Freeman et al. 2013, 2016).

Host trees exhibit varying responses to beetle infestation across different species, leading to noticeable distinctions. Staining, gumming, sugary exudate, and frass (excrement and sawdust) from holes that are about 0.85 mm in diameter on living trees can all be signs of the beetle’s boring activity. The abdomen of the female beetle can often be seen protruding out of the entrance hole through the bark, guarding the developing larvae within the galleries. Advanced beetle infestations and fungal infections on highly susceptible hosts (PSHB-reproductive hosts) will eventually lead to branch dieback and tree mortality. At least 161 tree species, 77 of them native, are known to be attacked in South Africa (FABI 2023). To date, 83 (41 of which are native) tree species have been identified as reproductive hosts including maples (*Acer* spp.), oaks (*Quercus* spp.), and willows (native and alien *Salix* spp.), native coral trees (*Erythrina* spp.) and bushwillows (*Combretum* spp.). PSHB also poses a threat to many economically important tree crops including avocado (*Persea americana*), apple (*Malus domestica*), and various stone fruit trees (*Prunus* spp.) (Jones and Paine 2017; de Jager and Roets 2022, 2023)—see Table S1 for a list of known reproductive and non-reproductive host species in South Africa.

PSHB poses a substantial risk to the agricultural, forestry, and urban and natural forest sectors throughout the country (Paap et al. 2018). A study by de Wit et al. (2022) used an ex-ante assessment to predict the cost of the PSHB invasion in South Africa by modelling the potential growth in PSHB populations and forecasting the impact on the net present cost resulting from damage to natural and urban forests, commercial forestry, and the avocado industry over a 10-year period (assuming unmitigated spread). The predicted net cost is 18.45 billion international dollars (Int. $), or 0.66% of South Africa’s GDP for the baseline scenario, with the bulk of the costs coming from the removal of infested urban trees.

### Study site

**Fig. 1:**
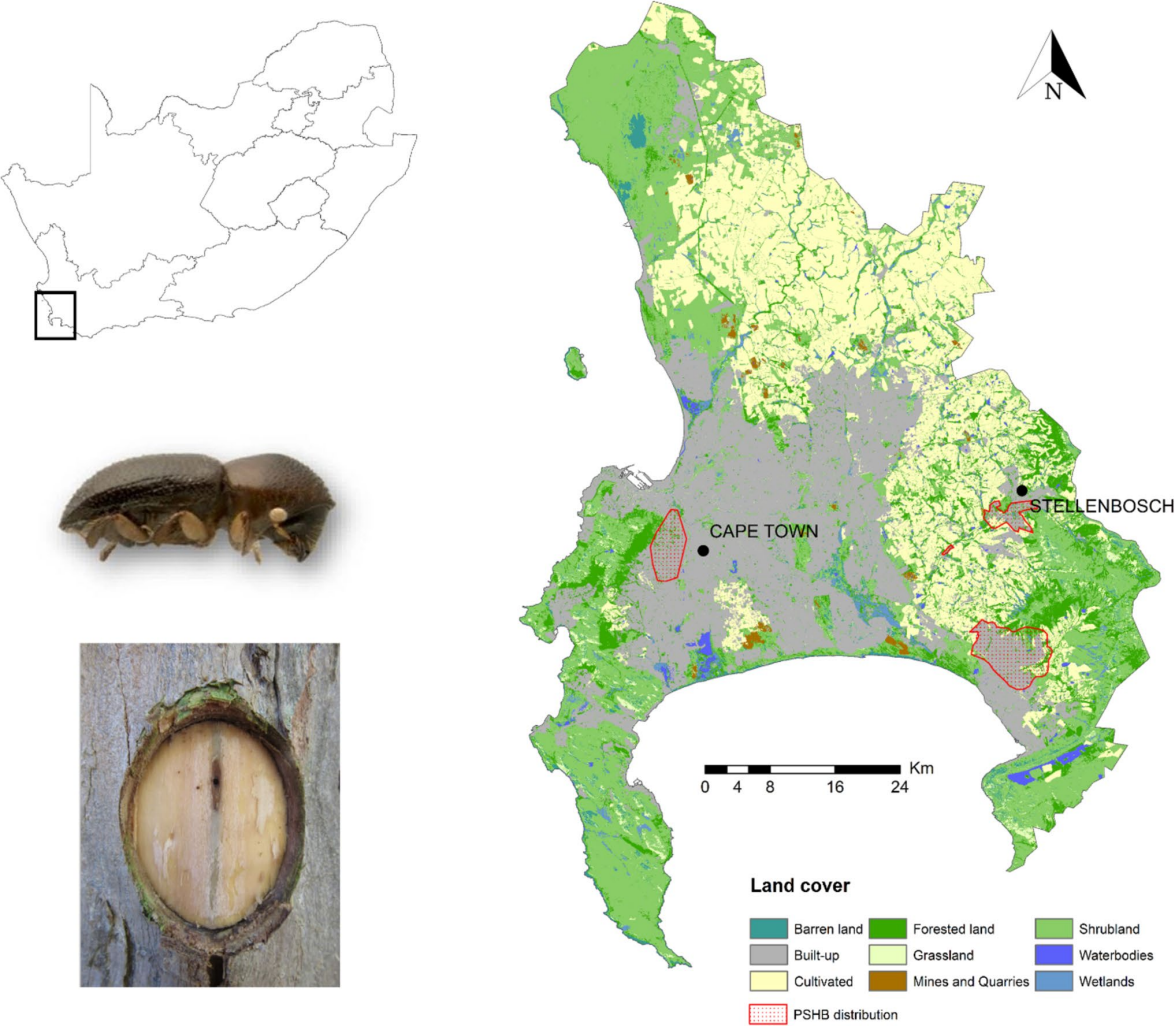
Location and land cover of the study area which comprises metropolitan Cape Town and the neighbouring town of Stellenbosch, South Africa (data: South African National Land Cover, Department of Forestry, Fisheries and the Environment 2020). The current known polyphagous shot hole borer distribution within the study area (data: City of Cape Town, updated 19 April 2023), the ambrosia beetle *Euwallacea fornicatus* and the entry hole and associated fungal stain are also shown

Our study area comprised two urban areas in South Africa’s Western Cape Province: Cape Town (33°55′31″S 18°25′26″E) and Stellenbosch (33°55′12″S 18°51′36″E). Both areas occur at the southwestern tip of Africa within the Cape Floristic Region (CFR), a global biodiversity hotspot (Fig. 1). Fynbos, a fire-prone and fire-adapted shrubland, is the dominant natural vegetation in the area, thriving on sandy and infertile soils (Cowling et al. 1996). The City of Cape Town alone contains 19 endemic vegetation types and over 190 endemic plant species (Holmes et al. 2012). Human population densities are high with 4.8 million and 196 000 people, respectively, in the City of Cape Town and the town of Stellenbosch (City of Cape Town 2021a; Department of Social Development 2021).

Due to strong participation by community members on platforms such as iNaturalist (https://www.inaturalist.org/) and aided by initiatives such as “The Great Southern Bioblitz” (https://www.inaturalist.org/projects/great-southern-bioblitz-2022-umbrella), the City of Cape Town and the town of Stellenbosch have comprehensive coverage of iNaturalist observations. These two adjacent areas have also recently experienced PSHB invasions (2019 and 2022 for Cape Town and Stellenbosch, respectively). There is also significant concern and interest in PSHB in these urban areas due to the importance of many highly susceptible (particularly non-native) tree species. For example, Stellenbosch is well known for the oak trees (*Quercus* sp.), some of which are highly susceptible PSHB-reproductive hosts, that have been extensively planted within and around the town (Donald 1978). This town is sometimes referred to as *Eikestad* (in Afrikaans), or Oak City (direct translation into English). Compared to Stellenbosch, the City of Cape Town has a relatively low tree canopy cover at just six percent (City of Cape Town 2021b). However, both Cape Town and Stellenbosch, the oldest and second oldest towns in South Africa, respectively, have been strongly influenced by European colonization, resulting in the introduction of various non-native species (Pooley 2018). As a result, a large proportion of the urban tree populations comprises non-native species such as *Acer negundo, Quercus robur*, and *Platanus* × *hispanica*. A high proportion of non-native tree species in the urban landscape can increase the probability of introduced pests and pathogens finding and establishing on suitable hosts (Colunga-Garcia et al. 2010).

To delineate the boundary of our study area, the boundary for each metropolitan area was first defined, then merged. For Cape Town’s boundary, the City of Cape Town metropolitan municipal boundary shapefile was obtained from the Municipal Demarcation Board (MDB 2022; updated 25 June 2019). To delineate the boundary for Stellenbosch, a combination of the municipal boundary and quaternary catchments was used. Spatial data for quaternary catchments of South Africa were obtained from the Department of Water and Sanitation (2011). Quaternary catchments are “hydrological units that are hierarchically nested from the primary drainage basin, through to secondary, tertiary, and quaternary level” (Nel et al. 2011). Using ArcGIS 10.7.1, those quaternary catchments which encompassed the urbanized parts of the town of Stellenbosch were selected, clipped, and merged into a single polygon. This polygon was then intersected with the Stellenbosch local municipal boundary polygon. The Cape Town and Stellenbosch boundaries were then merged into one final polygon delineating the study area (Fig. 1).

### Data collection and analyses

Occurrence data of PSHB-reproductive hosts within our study area were exported from the project titled “Reproductive hosts at risk of PSHB in South Africa” in iNaturalist (https://www.inaturalist.org/projects/reproductive-hosts-at-risk-of-pshb-in-south-africa; accessed 14 February 2023). Only observations with one or more agreements on identification were included. This project includes all species from the Forestry and Agricultural Biotechnology Institute’s most up-to-date PSHB reproductive host species list (https://www.fabinet.up.ac.za/images/PSHB/8-PSHB_host_list_2022-08-02.pdf; updated on 17 April 2023).

The movement of infested wood is a crucial pathway for the spread of PSHB. For example, the informal urban firewood trade (which serves as a vital energy source and contributes to income stability for numerous urban residents) and the trade in infested nursery material poses a significant risk for long-distance dispersal of PSHB, as firewood and nursery stock are transported over extensive distances, including into natural environments (de Wit et al. 2022). The disposal of infested plant biomass at formal (and informal) waste facilities without following appropriate handling and phytosanitary measures, also presents a major dispersal pathway for PSHB (van Rooyen et al. 2021).

Spatial data for various plant biomass sites were obtained from the City of Cape Town Open Data Portal (https://odp-cctegis.opendata.arcgis.com/) and identified through consultation with local arborists and municipal workers, and our own observations. The sites identified, however, do not represent all plant biomass sites within the study area. For example, many informal dumping sites, firewood distributors and nurseries occur across the study area for which no data currently exist. However, we are confident that the sites included in our analyses provide an appropriate sample to draw reliable conclusions on where best to monitor PSHB infestations. Moreover, the approach developed in this study allows for the seamless integration of these data as they become available—the spatial analyses can be rerun to provide updated maps of priority PSHB monitoring areas.

The Box Elder (*Acer negundo*) has been identified as one of the most susceptible species to PSHB in South Africa and abroad (e.g. agric.wa.gov.au/borer; FR unpublished data). These “amplifier” trees significantly enhance propagule pressure thereby increasing the risk of future impacts on nearby trees. This, combined with its relative abundance in South African urban tree plantings, the rapid onset of dieback symptoms after infestation and the relative ease in which initial symptoms can be detected, make this species an ideal candidate as sentinel species for the detection of PSHB infestations. As a result, occurrence data for *A. negundo* trees within our study area were exported from the iNaturalist project titled “Acer negundo in South Africa” (https://www.inaturalist.org/projects/acer-negundo-in-south-africa; Accessed 14 February 2023). Given that relatively few observations of this species have been recorded within our study area, all records (including those with no supporting identifications) were retained for the analysis. This resulted in 349 records for *A. negundo* of which 22% had no identification agreements. As this species does not pose identification challenges, we consider this approach to be appropriate.

iNaturalist records for PSHB reproductive hosts and *A. negundo* were mapped separately using ArcGIS 10.7.1. These records were clipped to our study area boundary. All PSHB reproductive host records included observations of *A. negundo* but given the importance of *A. negundo* as a sentinel species for monitoring and surveillance, they were mapped separately. A grid (1 × 1 km cell size) covering the extent of the study area was then created using the Create Fishnet geoprocessing tool. A Spatial Join analysis was then used to join the grid to the PSHB reproductive hosts observation shapefile. This was also done separately for the *A. negundo* shapefile. This produced datasets of densities (records per square kilometre) for PSHB reproductive host trees and *A. negundo* for the study area. Each grid polygon was then converted to a raster dataset and reclassified.

The Near analysis was used to calculate the distance from each grid cell to the nearest plant biomass sites. Priorities were then assigned based on PSHB reproductive host and *A. negundo* densities (per km^2^) and proximity to plant biomass sites. To provide more detailed operational guidance for practitioners, roads within priority grid cells were identified to determine which roads should be prioritised for visual surveys. Spatial data for roads within the study area were obtained from the City of Cape Town data portal and Humanitarian Data Exchange. These shapefiles were merged and clipped to our study area. A Spatial Join analysis was used to join the road shapefile with the PSHB reproductive host densities (with priorities). This was also done separately for the *A. negundo* shapefile.

## Results

Our monitoring protocol, which combined citizen science data on species occurrence and spatial data on urban facilities, identified priority areas which require monitoring for the early detection and management of a highly damaging pest-pathogen complex. Using the invasion of PSHB in two urban centres in the Western Cape, South Africa as case study, priority monitoring areas were determined based on PSHB reproductive host density (using data from iNaturalist) and their proximity to plant biomass sites.

**Fig. 2:**
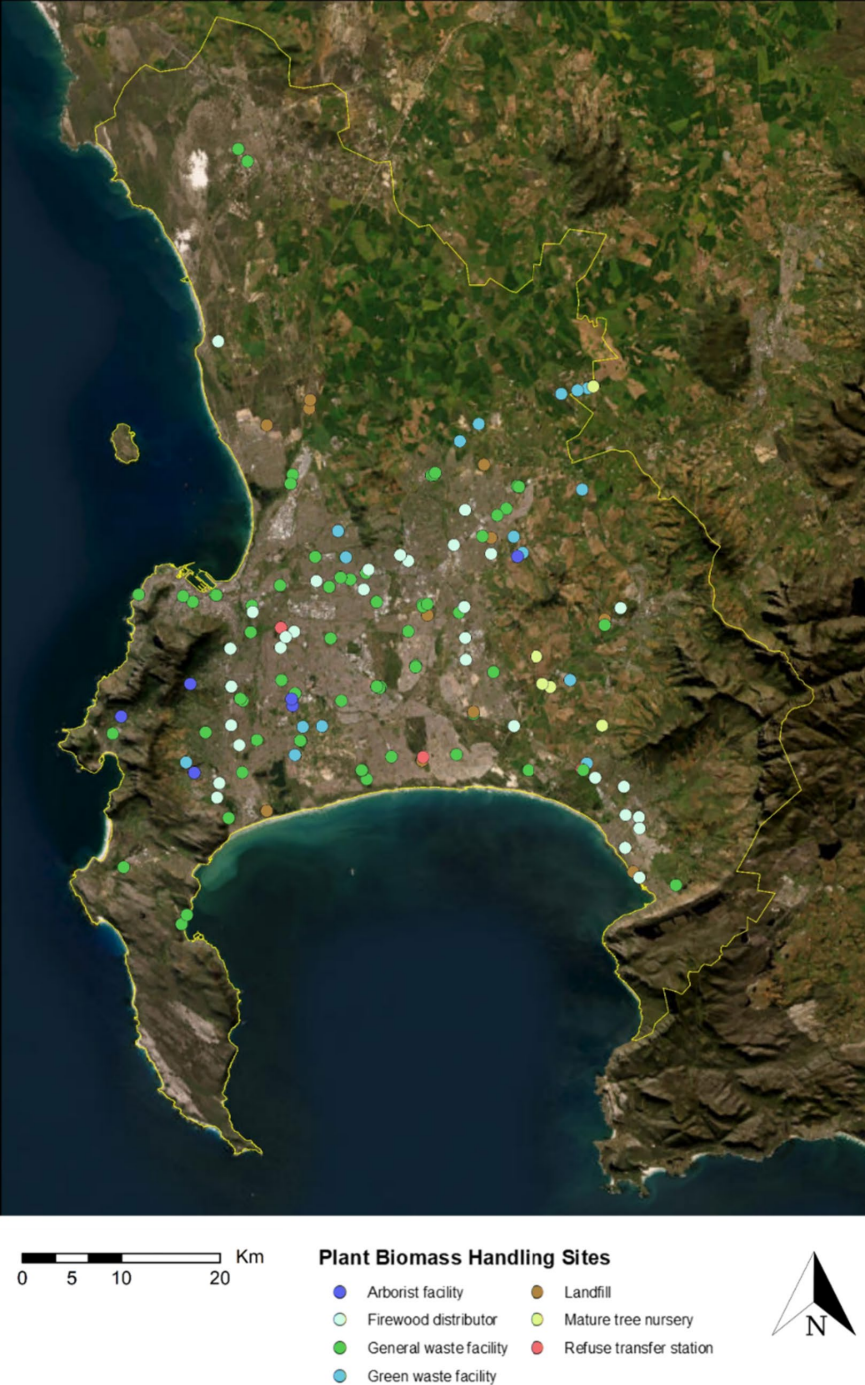
The location of plant biomass sites across the study area. Data from the City of Cape Town Open Data Portal (Accessed 22 March 2023), and through consultation with local arborists and municipal workers

We identified and mapped 140 plant biomass sites across the study area (Fig. 2). These included arborist facilities, firewood distributors, general waste facilities, green waste facilities, landfills, mature tree nurseries, and refuse transfer stations. A total of 8654 records for 55 PSHB reproductive host taxa, and 349 for *A. negundo* were included in our analyses. The highest number of observations for PSHB reproductive hosts in our study area include *Psoralea pinnata* (1330, native), *Kiggelaria* a*fricana* (1101, native), *Acacia longifolia* (875, non-native), *Acacia mearnsii* (720, non-native), and *Acacia melanoxylon* (585, non-native). Species with the most observations in the ten highest priority areas (km^2^) for PSHB monitoring include *Psoralea pinnata* (272), *Acacia longifolia* (173), *Quercus robur* (70, non-native), *Acacia melanoxylon* (52), and *Kiggelaria africana* (46). These areas all occur within one kilometre of plant biomass sites (see Table S2).

**Fig. 3:**
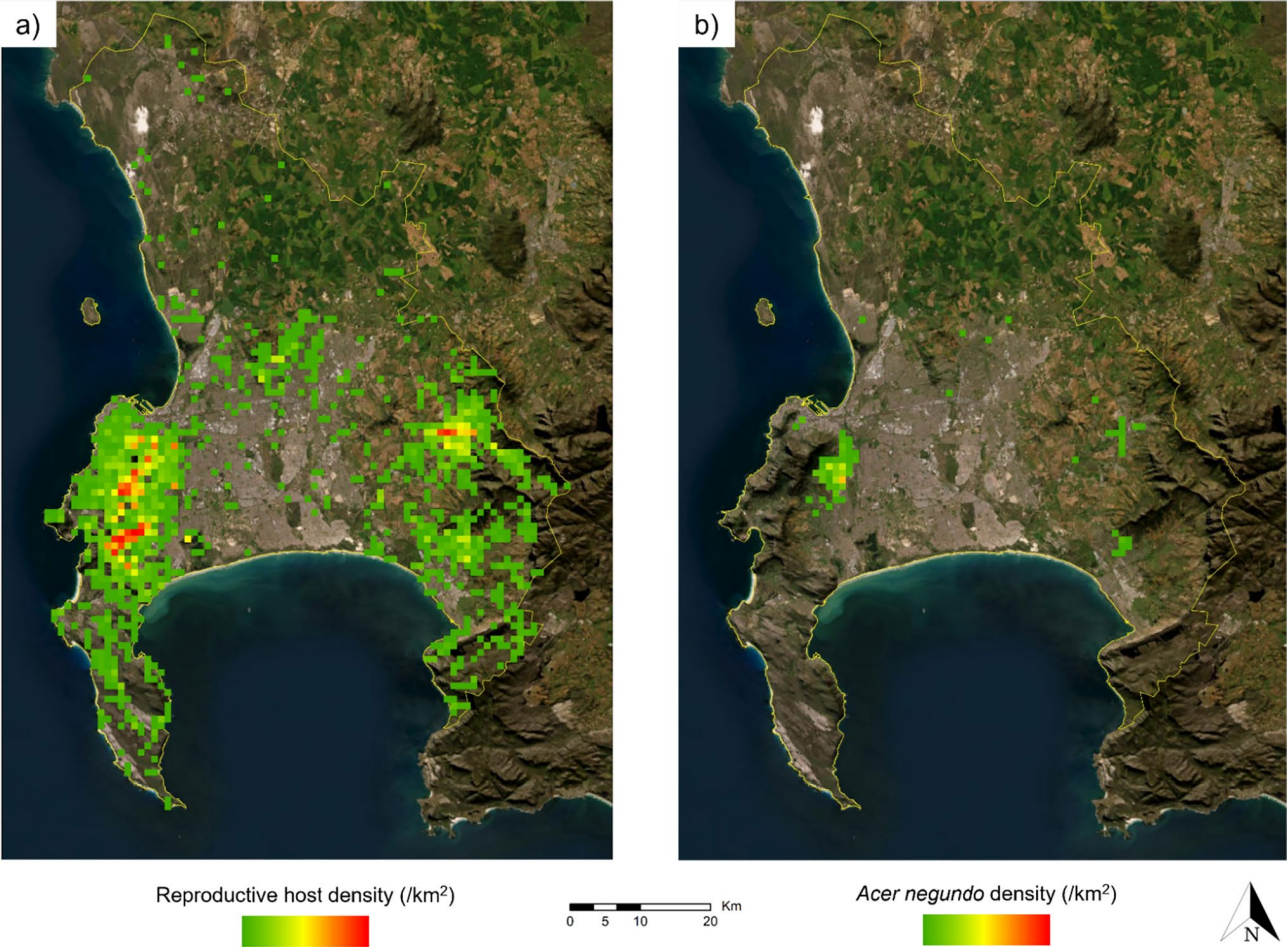
Densities (number of species’ observations per km^2^ grid cell) for a) all polyphagous shot hole borer (PSHB) reproductive hosts (*n* = 8654), and b) *Acer negundo* (*n* = 349), a highly susceptible reproductive host. Red = high density, green = low density. Data from iNaturalist (https://www.inaturalist.org/projects/reproductive-hosts-at-risk-of-pshb-in-south-africa” and https://www.inaturalist.org/projects/acer-negundo-in-south-africa, Accessed 14 February 2023)

**Fig. 4:**
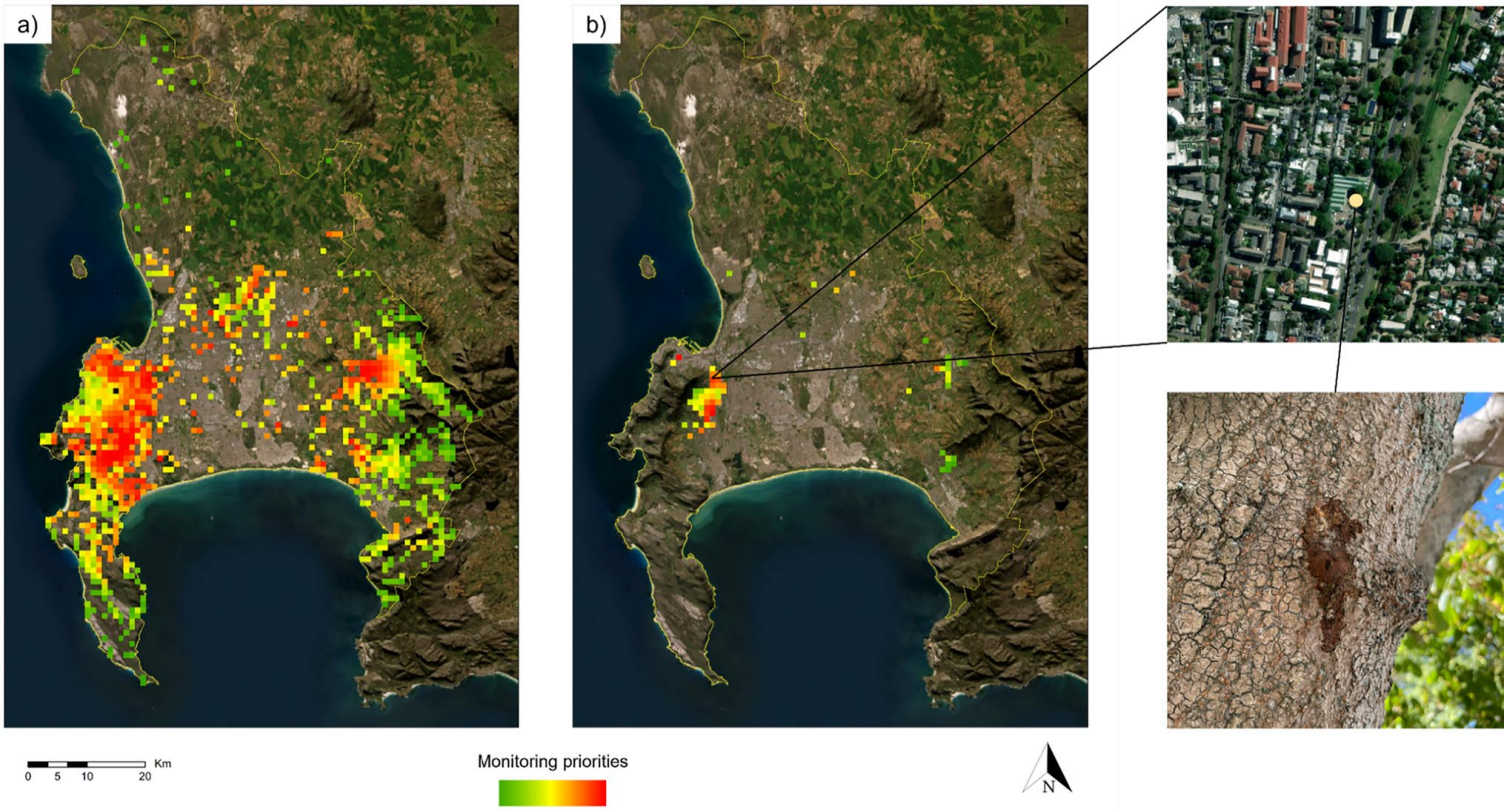
Priority monitoring areas (1km^2^ grid cells) based on polyphagous shot hole borer (PSHB) reproductive host density and their proximity to plant biomass sites for **a** all reproductive hosts, and **b** *Acer negundo*. Red = high priority, green = low priority. A visual survey of a high priority *Acer negundo* monitoring site identified in our protocol detected a new PSHB infestation outside a nursery (photo credit: LJ Potgieter, 18 February 2023). Data from iNaturalist (https://www.inaturalist.org/projects/reproductive-hosts-at-risk-of-pshb-in-south-africa and https://www.inaturalist.org/projects/acer-negundo-in-south-africa, Accessed 14 February 2023)

High-priority areas for PSHB monitoring include those areas with the highest density of reproductive hosts (Fig. 3) close to plant biomass sites. These areas include Cape Town's Central Business District (CBD) and the southern suburbs which lie on the north- and south-eastern slopes of Table Mountain, respectively. Other high-priority areas include most of the urbanized parts of Stellenbosch and dozens of isolated areas in the central and south-western parts of the study area (Fig. 4a).

High priority areas for PSHB monitoring based on the distribution of the highly susceptible, amplifier species *A. negundo*, include the southern suburbs on the north-eastern slopes of the Table Mountain National Park (Fig. 4b). Other high-priority areas include isolated areas in Cape Town’s CDB and in the town of Stellenbosch. A recent visual survey of a high-priority *Acer negundo* monitoring site identified in our protocol detected a new PSHB infestation located outside a nursery (Fig. 4b).

Lower priority areas occur in the mountainous parts along the south-western (Cape Peninsula) and south-eastern parts of the study area (Fig. 4). Protected Areas such as the Table Mountain National Park (south-west) and Steenbras Nature Reserve (south-east) make up a significant proportion of these areas. The PSHB reproductive host densities are relatively low and their proximity to plant biomass sites is high. Data deficient (few to no iNaturalist observations) areas include agricultural areas in the north-eastern parts of the study area and the Cape Flats in the central region of the study area. While these areas are characterized by low tree canopy cover and therefore low PSHB reproductive host densities, few of the trees which do occur have been captured in iNaturalist (see Discussion for further details).

Our spatial monitoring prioritisation approach also identified high-priority roads for visual surveys (see Table S2 and 3). This provides practical and operational guidance for practitioners conducting the monitoring. In addition to visual road surveys, our plant biomass site map also serves as a useful guide for site-specific monitoring for PSHB infestations (see Table S4).

## Discussion

Monitoring urban biological invasions is critical for preventing the establishment and spread of invasive species, protecting natural resources, minimizing negative impacts on biodiversity and ecosystem services, and ensuring the effectiveness of management efforts (Persad and Tobin 2015). However, there are generally a lack of robust and reliable baseline monitoring data to effectively guide management action (Lindenmayer et al. 2013), especially in urban areas (Potgieter et al. 2021). The increased adoption of citizen science platforms like iNaturalist has resulted in an unprecedented abundance of data on species occurrences in urban areas (Spear et al. 2017). Recent studies have emphasised the demand for detection and management tools, and particularly the integration of citizen science into official pest and pathogen monitoring programmes (e.g. Green et al. 2023).

This study used citizen science data to develop a spatially explicit prioritisation protocol for monitoring pests/pathogens in urban areas, using PSHB invasions in two urban centres in the Western Cape, South Africa, as a case study. It shifts the focus away from ad hoc monitoring approaches and provides urban conservation practitioners with guidance on where to focus monitoring efforts to detect new or expanding infestations.

Priority monitoring areas were determined based on PSHB reproductive host density (using species occurrence data from iNaturalist) and their proximity to plant biomass sites (Fig. 4). Roads within priority grid cells were also identified to provide urban practitioners with guidance on which roads should be prioritised for visual surveys and baited traps (Table S2 and 3). Priority monitoring areas were also identified using the distribution of *A. negundo*, a highly susceptible PSHB reproductive host that can serve as sentinel species for the detection of PSHB infestations. These priority monitoring areas proved crucial in detecting new infestations in our study area—a recent visual survey of a high-priority monitoring site identified in our protocol detected a new PSHB infestation on an *A. negundo* tree located outside a nursery (Fig. 4b). This tree has already been felled as per the City of Cape Town’s PSHB management protocol. Our monitoring protocol also allows for the assessment of the effectiveness of control measures implemented such as the removal of highly infested reproductive host trees. By monitoring susceptible reproductive host trees and plant biomass sites close to an initial infestation, it is possible to determine whether control measures are having the desired effect (slowing the rate of spread or reducing propagule pressure) and to adjust management strategies if necessary.

While visual tree surveys by trained monitors are a more accurate and precise way of detecting infestations, deploying monitoring traps at priority sites can also be used. Monitoring traps involve the use of an attractant to bait and capture pests for detection purposes. Although costly, a surveillance trapping protocol could lead to earlier detection of newly established urban tree pests and pathogens or to track the expansion of existing infestations, thereby increasing the probability of achieving successful eradication and mitigating negative impacts (Epanchin-Niell et al. 2014). The monitoring priorities identified in this study can be used to determine where to place monitoring traps. For PSHB traps, Quercivorol (a plant-based chemical lure), can be used to attract PSHB if a beetle is within the local area (Dodge et al. 2017). For example, traps with Quercivorol lures can be placed at intervals along and/or beyond the leading edge of the infestation, targeting high-priority sites. *α*-Copaene, another potential attractant for PSHB, shows equivalent efficacy to Quercivorol, but using both lures captures significantly more PSHB than either lures alone (Kendra et al. 2017). Traps can also be deployed at plant biomass sites such as landfills, nurseries, green waste processing facilities, and firewood storage and distribution lots. If traps are successful in capturing a pest, depending on the number and location of traps, the relevant management authority can initiate a visual survey to delimit the extent of the infestation.

Effective management of invasive pests in urban areas requires a multidisciplinary approach, involving collaboration between scientists, policymakers, and the public. Monitoring invasive species can raise awareness among the public and stakeholders about the importance of preventing the spread of invasive pests and pathogens, and the need for collaborative management efforts (Gallo and Waitt 2011). Using citizen science data in identifying monitoring priorities increases transparency and therefore public trust in management interventions (Cardoso et al. 2017). This approach can also help raise awareness and encourage action by fostering a sense of ownership and responsibility among participants.

The integration of citizen science with emerging technologies has great promise for conservation management. Many technologies are now being used by professionals for the detection and monitoring of forest pests (de Groot et al. 2023). For example, unmanned aerial vehicles (UAVs) and satellites can provide high-resolution imagery which can enhance the accuracy and granularity of community-contributed data. Equipped with various sensors, including cameras and LiDAR, these data can enable fine-scale monitoring of individual urban trees, canopy structures, and vegetation health. Integrating these data sources with citizen science efforts can enhance the robustness of urban forest monitoring.

### Limitations

There are also several limitations to using community science data. iNaturalist observations are submitted voluntarily by users, so the data collected might not be representative of the entire population of a particular species. For example, certain taxa might be more popular among iNaturalist users than others, leading to sampling bias (Di Cecco et al. 2021). While iNaturalist has mechanisms in place to verify observations, such as peer review and expert identification, there is still the potential for misidentification or errors in data entry (Crall et al. 2010). This can result in inaccurate data being collected and shared on the platform. Volunteer contributors also tend have more spatially and temporally concentrated sampling effort compared to professional sampling schemes (Boakes et al. 2010). Locations with more species’ observations are also those with higher numbers of users on the platform. The greater the number of people accessing the application, the greater the amount of available information, generating a possible observation bias.

Participation in citizen science does not always reflect the demographics of the population. Individuals from historically underrepresented groups are less likely to participate resulting in a high proportion of affluent participants (NASEM 2018). For example, in our study area there are a lack of data (few to no iNaturalist observations) in the low-lying areas known as the Cape Flats which comprises mostly low-income townships and informal settlements. While these areas have low tree canopy cover and therefore have low densities of PSHB reproductive hosts, most of the residents here do not have the capacity to capture citizen science data (e.g. Anderson and O’Farrell 2012; Potgieter et al. 2019). As a result, few of the trees which do occur have been captured in iNaturalist. Despite these limitations, iNaturalist provides data in sufficient quantity and quality for research and management applications.

Additional limitations include taxonomic identification challenges based on morphological features (some taxa can only be accurately identified through DNA barcoding), and destructive sampling might be necessary to identify some taxa. Also, citizen scientists might not get access to some urban areas e.g. alongside train tracks or within military bases.

## Conclusion

We have developed a spatially explicit prioritisation protocol for monitoring invasive pests/pathogens in urban areas. This monitoring approach can be a critical component of any Integrated Pest Management program, as it allows for early detection of pest infestations and provides valuable data for making informed management decisions.

Utilizing citizen science offers a cost-effective approach for gathering crucial monitoring data while fostering significant citizen engagement and can play a pivotal role in the adaptive management learning process. This study highlights the value of citizen science data in informing the management of urban biological invasions. By leveraging the power of crowdsourcing and overcoming many of the challenges of traditional survey methods, iNaturalist should be considered an essential tool for conservation monitoring in urban landscapes.

## Author contributions

All authors contributed to the study conception and design. Material preparation, data collection and analysis were performed by LJP. The first draft of the manuscript was written by LJP, and all authors commented on previous versions of the manuscript. All authors read and approved the final manuscript.

## Supplementary Information

Below is the link to the electronic supplementary material.

Supplementary file1 (DOCX 220 kb)

## Data Availability

The datasets generated during and/or analysed during the current study are available from the corresponding author on reasonable request. Various datasets used during this study are publicly accessible and made available under the City of Cape Town’s Open Data Portal, policy no. 27781 (https://odp-cctegis.opendata.arcgis.com/).
