# Supplementary material for: Monitoring urban biological invasions using citizen science: the polyphagous shot hole borer (Euwallacea fornicatus)
Source: J Pest Sci (2004). 2024 Jan 27;97(4):2073–85. doi: 10.1007/s10340-024-01744-7 (PMC11420376; doi:10.1007/s10340-024-01744-7)
Supplement: Supplementary file 1 — Supplementary file1 (DOCX 220 kb) [file 10340_2024_1744_MOESM1_ESM.docx]

# Supplementary information

## **Table S1**. List of confirmed host plants of the polyphagous shot hole borer (*Euwallacea* *fornicatus*) beetle in South Africa (as per FABI 2023, updated 17 April 2023). The list includes only those taxa from which its primary fungal symbiont, *Fusarium euwallaceae*, or a beetle individual was successfully isolated, and its identity confirmed using DNA sequence fingerprinting. Reproductive hosts include only those hosts that have been shown to be suitable for PSHB reproduction at least once. Non-reproductive hosts include those from which either PSHB or *F. euwallaceae* was isolated, but no evidence of PSHB reproduction has been verified. Importantly, this list serves as a guideline as some non-reproductive hosts can become reproductive under certain environmental conditions (Paap et al. 2020). *Regarded as highly susceptible reproductive host species. Refer to FABI (2023) for an updated list of host trees (<https://www.fabinet.up.ac.za/index.php/research-groups/pshb-new/background-info/host-trees>).

| **REPRODUCTIVE HOSTS** | | | | |
| --- | --- | --- | --- | --- |
| **Scientific name** | **Common name** | **Family name** | **Invasion status** | **NEMBA category** |
| *Acacia longifolia* | Long-leaved wattle | Fabaceae | Invasive alien | 1b |
| *Acacia mearnsii* | Black wattle | Fabaceae | Invasive alien | 2 |
| *Acacia melanoxylon* | Blackwood | Fabaceae | Invasive alien | 2 |
| *Acalypha glabrata* | Forest false nettle | Euphorbiaceae | Native |  |
| *Acer buergerianum* | Trident (Chinese) maple | Sapindaceae | Invasive alien | 3/- |
| *Acer negundo** | Boxelder | Sapindaceae | Invasive alien | 3/- |
| *Acer palmatum* | Japanese maple | Sapindaceae | Alien |  |
| *Acer pseudoplatanus* | Sycamore maple | Sapindaceae | Alien |  |
| *Acer saccharinum* | Silver maple | Sapindaceae | Alien |  |
| *Afrocarpus falcatus* | Outeniqua yellowwood | Podocarpaceae | Native |  |
| *Afzelia quanzensis* | Pod mahogany | Fabaceae | Native |  |
| *Allophylus natalensis* | Dune false crowberry | Sapindaceae | Native |  |
| *Anisodontea scabrosa* | Rough-leaf African mallow | Malvaceae | Native |  |
| *Baphia racemosa* | Violet pea | Fabaceae | Native |  |
| *Bauhinia galpinii* | Pride of de Kaap | Fabaceae | Native |  |
| *Bauhinia variegata* | Orchid tree | Fabaceae | Invasive alien | 1b/3 |
| *Brachychiton discolor* | Pink flame tree | Malvaceae | Alien |  |
| *Brachylaena discolor* | Coast silver oak | Malvaceae | Native |  |
| *Calodendrum capense* | Cape chestnut | Rutaceae | Native |  |
| *Calpurnia aurea* | Wild laburnum | Fabaceae | Native |  |
| *Carya illinoinensis* | Pecan nut | Juglandaceae | Alien |  |
| *Cassia fistula* | Golden shower tree | Fabaceae | Alien |  |
| *Casuarina cunninghamiana* | Beefwood | Casuarinaceae | Invasive alien | 1b/2 |
| *Combretum erythrophyllum* | River bushwillow | Combretaceae | Native |  |
| *Combretum kraussii* | Forest bushwillow | Combretaceae | Native |  |
| *Cussonia spicata* | Cabbage tree/Kiepersol | Araliaceae | Native |  |
| *Dichrostachys cinerea* | Sickle bush | Fabaceae | Native |  |
| *Diospyros dichrophylla* | Star apple | Ebenaceae | Native |  |
| *Diospyros glabra* | Cape star-apple | Ebenaceae | Native |  |
| *Diospyros whyteana* | Bladdernut | Ebenaceae | Native |  |
| *Erythrina caffra* | Coast coral tree | Fabaceae | Native |  |
| *Erythrina lysistemon* | Common coral tree | Fabaceae | Native |  |
| *Ficus natalensis* | Natal fig | Moraceae | Native |  |
| *Ficus trichopoda* | Swamp fig | Moraceae | Native |  |
| *Gleditsia triacanthos* | Honey locust | Fabaceae | Invasive alien | 1b |
| *Grewia occidentalis* | Cross berry | Malvaceae | Native |  |
| *Halleria lucida* | Tree fuchsia | Stilbaceae | Native |  |
| *Harpephyllum caffrum* | Wild plum | Anacardiaceae | Native |  |
| *Indigofera jucunda* | River indigo | Fabaceae | Native |  |
| *Kiggelaria africana* | Wild peach | Achariaceae | Native |  |
| *Koelreuteria paniculata* | Golden raintree | Sapindaceae | Alien |  |
| *Liquidambar styraciflua* | Liquidambar/Sweetgum | Altingiaceae | Alien |  |
| *Loxostylis alata* | Wild pepper tree | Anacardiaceae | Native |  |
| *Maesa lanceolata* | False assegai | Primulaceae | Native |  |
| *Magnolia grandiflora* | Southern magnolia | Magnoliaceae | Alien |  |
| *Malus domestica* | Apple | Rosaceae | Alien |  |
| *Olea europaea* subsp*. africana* | Wild olive | Oleaceae | Native |  |
| *Persea americana* | Avocado | Lauraceae | Alien |  |
| *Photinia x fraseri* | Christmas berry | Rosaceae | Alien |  |
| *Platanus x acerifolia* | London plane | Platanaceae | Alien |  |
| *Podalyria calyptrata* | Water blossom pea | Fabaceae | Native |  |
| *Populus alba* | White poplar | Salicaceae | Invasive alien | 2 |
| *Populus nigra* | Lombardy poplar | Salicaceae | Alien |  |
| *Populus simonii* | Chinese cottonwood | Salicaceae | Alien |  |
| *Populus x canescens* | Grey poplar | Salicaceae | Invasive alien | 2 |
| *Prunus armeniaca* | Apricot | Rosaceae | Alien |  |
| *Prunus cerasifera* | Cherry plum | Rosaceae | Alien |  |
| *Prunus dulcis* | Almond | Rosaceae | Alien |  |
| *Prunus nigra* | Black plum | Rosaceae | Alien |  |
| *Psoralea affinis* | Tall fountain bush | Fabaceae | Native |  |
| *Psoralea aphylla* | Leafless fountain bush | Fabaceae | Native |  |
| *Psoralea pinata* | Fountain bush | Fabaceae | Native |  |
| *Pyrus communis* | Pear | Rosaceae | Alien |  |
| *Quercus palustris* | Pin oak | Fagaceae | Alien |  |
| *Quercus robur** | English oak | Fagaceae | Alien |  |
| *Quercus suber* | Cork oak | Fagaceae | Alien |  |
| *Rhamnus prinoides* | Shiny-leaf buckthorn | Rhamnaceae | Native |  |
| *Ricinus communis** | Castor bean | Euphorbiaceae | Invasive alien | 2 |
| *Robinia pseudoacacia* | Black locust | Fabaceae | Invasive alien | 1b |
| *Rosa setigera* | Climbing rose | Rosaceae | Alien |  |
| *Salix alba* | White willow | Salicaceae | Alien |  |
| *Salix babylonica** | Weeping willow | Salicaceae | Alien |  |
| *Salix mucronata* | Cape willow | Salicaceae | Native |  |
| *Senna mulitjuga* | November shower | Fabaceae | Alien |  |
| *Sparrmannia africana* | African hemp | Malvaceae | Native |  |
| *Sterculia murex* | Lowveld chestnut | Malvaceae | Native |  |
| *Trema orientalis* | Pigeon wood | Cannabaceae | Native |  |
| *Trichilia emetica* | Natal mahogany | Meliaceae | Native |  |
| *Ulmus parvifolia* | Chinese elm | Ulmaceae | Alien |  |
| *Vepris lanceolata* | White ironwood | Rutaceae | Native |  |
| *Viburnum odoratissimum* | Sweet viburnum | Adoxaceae | Alien |  |
| *Virgilia oroboides* subsp*. ferruginea* | Keurboom | Fabaceae | Native |  |
| *Wisteria sinensis* | Chinese wisteria | Fabaceae | Alien |  |
| **NON-REPRODUCTIVE HOSTS** | | | | |
| **Scientific name** | **Common name** | **Family name** | **Invasion status** | **NEMBA category** |
| *Acacia saligna* | Port Jackson willow | Fabaceae | Invasive alien | 1b |
| *Adansonia digitata* | Baobab | Malvaceae | Native |  |
| *Albizia adianthifolia* | Flat crown | Fabaceae | Native |  |
| *Allophylus decipiens* | Bastard taaibos | Sapindaceae | Native |  |
| *Bauhinia purpurea* | Butterfly orchid tree | Fabaceae | Invasive alien | 1b/3 |
| *Betula pendula* | Silver birch | Betulaceae | Alien |  |
| *Bougainvillea* sp*.* | Bougainvillea | Nyctaginaceae | Alien |  |
| *Buddleja saligna* | False olive | Scrophulariaceae | Native |  |
| *Camellia japonica* | Common camellia | Theaceae | Alien |  |
| *Cananga odorata* | Ylang Ylang | Annonaceae | Alien |  |
| *Ceiba pentandra* | Kapok | Malvaceae | Alien |  |
| *Ceiba speciosa* | Silk floss tree | Malvaceae | Alien |  |
| *Celtis africana* | White stinkwood | Cannabaceae | Native |  |
| *Chaetacme aristata* | Thorny elm | Ulmaceae | Native |  |
| *Cinnamomum camphora* | Camphor | Lauraceae | Invasive alien | 1b/3 |
| *Citrus limon* | Lemon | Rutaceae | Alien |  |
| *Citrus sinensis* | Orange | Rutaceae | Alien |  |
| *Cola natalensis* | Coshwood | Malvaceae | Native |  |
| *Commiphora harveyi* | Copper stem corkwood | Burseraceae | Native |  |
| *Cordia caffra* | Septee tree | Boraginaceae | Native |  |
| *Cordia myxa* | Assyrian plum | Boraginaceae | Alien |  |
| *Diospyros kaki* | Persimmon | Ebenaceae | Alien |  |
| *Diospyros lycoides* | Monkey plum | Ebenaceae | Native |  |
| *Dombeya rotundifolia* | Wild pear | Malvaceae | Native |  |
| *Dovyalis caffra* | Kei apple | Salicaceae | Native |  |
| *Ekebergia capensis* | Cape ash | Meliaceae | Native |  |
| *Eriobotrya japonica* | Loquat | Rosaceae | Invasive alien | 1b/- |
| *Erythrina livingstoniana* | Aloe coral tree | Fabaceae | Alien |  |
| *Eucalyptus camaldulensis* | River red gum | Myrtaceae | Invasive alien | 1b/2 |
| *Ficus carica* | Common fig | Moraceae | Alien |  |
| *Ficus sur* | Cape fig | Moraceae | Native |  |
| *Fraxinus americana* | American ash | Oleaceae | Invasive alien | 3/- |
| *Fraxinus excelsior* | European ash | Oleaceae | Alien |  |
| *Gymnosporia buxifolia* | Spike thorn | Celestraceae | Native |  |
| *Hakea salicifolia* | Willow-leaved hakea | Proteaceae | Invasive alien | 1b/- |
| *Hibiscus rosa-sinensis* | Hibiscus | Malvaceae | Alien |  |
| *Ilex mitis* | Cape holly | Aquifoliaceae | Native |  |
| *Jacaranda mimosifolia* | Jacaranda | Bignoniaceae | Invasive alien | 1b/- |
| *Leonotis leonurus* | Wild tobacco | Lamicaeae | Native |  |
| *Macadamia sp.* | Macadamia nut | Proteaceae | Alien |  |
| *Melia azedarach* | Syringa | Meliaceae | Invasive alien | 1b/3 |
| *Melianthus major* | Honey flower | Francoaceae | Native |  |
| *Metasequoia glyptostroboides* | Dawn redwood | Cupressaceae | Alien |  |
| *Monoon longifolium* | False ashoka | Annonaceae | Alien |  |
| *Morus nigra* | Black mulberry | Moraceae | Alien |  |
| *Nuxia floribunda* | Forest elder | Stilbaceae | Native |  |
| *Olea europaea* subsp*. europaea* | Cultivated olive | Oleaceae | Alien |  |
| *Olinia ventosa* | Hard pear | Penaeaceae | Native |  |
| *Osteospermum moniliferum* | Bietou | Asteraceae | Native |  |
| *Platanus occidentalis* | American plane | Platanaceae | Alien |  |
| *Platanus racemosa* | Californian plane | Platanaceae | Alien |  |
| *Plumeria rubra* | Frangipani | Apocynaceae | Alien |  |
| *Podocarpus henkelii* | Henkel’s yellowwood | Podocarpaceae | Native |  |
| *Protea mundii* | Forest sugar bush | Proteaceae | Native |  |
| *Prunus africana* | Red stinkwood | Rosaceae | Native |  |
| *Prunus avium* | Sweet cherry | Rosaceae | Alien |  |
| *Prunus persica* | Peach | Rosaceae | Alien |  |
| *Psidium guajava* | Guava | Myrtaceae | Invasive alien | 2/3 |
| *Quercus nigra* | Water oak | Fagaceae | Alien |  |
| *Quercus rugosa* | Net leaf oak | Fagaceae | Alien |  |
| *Rapanea melanophloeos* | Cape beech | Primulaceae | Native |  |
| *Schinus molle* | Pepper tree | Anacardiaceae | Alien |  |
| *Schotia brachypetala* | Weeping boerbean | Fabaceae | Native |  |
| *Sclerocarya birrea* | Marula | Anacardiaceae | Native |  |
| *Searsia chirindensis* | Red currant | Anacardiaceae | Native |  |
| *Searsia lansea* | Karree | Anacardiaceae | Native |  |
| *Senegalia (Acacia) burkei* | Black monkey-thorn | Fabaceae | Native |  |
| *Senegalia (Acacia) galpinii* | Monkey-thorn | Fabaceae | Native |  |
| *Solanum mauritianum* | Bugweed | Solonaceae | Invasive alien | 1b |
| *Stenocarpus sinuatus* | Firewheel tree | Proteaceae | Alien |  |
| *Syzygium cordatum* | Waterberry | Myrtaceae | Native |  |
| *Taxodium distichum* | Swamp cypress | Cupressaceae | Alien |  |
| *Ulmus minor = procera* | English elm | Ulmaceae | Alien |  |
| *Vachellia (Acacia) karroo* | Sweet thorn | Fabaceae | Native |  |
| *Vachellia (Acacia) sieberiana* var. *woodii* | Paper bark thorn | Fabaceae | Native |  |
| *Virgilia divaricata* | Keurboom | Fabaceae | Native |  |
| *Vitis vinifera* | Grape vine | Vitaceae | Alien |  |
| *Xylotheca kraussiana* | African dog rose | Achariaceae | Native |  |

## **Table S2**. Roads per priority km^2^ grid cell for polyphagous shot hole borer (*Euwallacea* *fornicatus*) visual surveys based on reproductive host densities (per km^2^) and their proximity to plant biomass sites (PBS). “N/A” = no roads within the km^2^ grid cell.

| **Priority km^2^ grid cell** | **Reproductive host/km^2^** | **Distance to nearest PBS (m)** | **Road name** | **Jurisdiction** | **Latitude** | **Longitude** |
| --- | --- | --- | --- | --- | --- | --- |
| 1 | 225 | 0 | Orpen Road | Cape Town | -34,054583 | 18,431992 |
| 2 | 68 | 53 | Klipfontein Road | Cape Town | -33,954514 | 18,481391 |
| 3 | 106 | 313 | Orpen Road | Cape Town | -34,05462 | 18,41969 |
| 4 | 58 | 43 | Kromboom Parkway | Cape Town | -33,996837 | 18,483191 |
| 5 | 77 | 316 | Orpen Road | Cape Town | -34,061205 | 18,424326 |
| 6 | 77 | 445 | Tokai Road | Cape Town | -34,061944 | 18,423889 |
| 7 | 155 | 687 | Soetvlei Avenue | Cape Town | -34,05452 | 18,433814 |
| 8 | 23 | 0 | Santhagen | Stellenbosch | -33,937733 | 18,827609 |
| 9 | 30 | 566 | Zomerlust Avenue | Cape Town | -34,034402 | 18,44185 |
| 10 | 93 | 969 | Firgrove Way | Cape Town | -34,042346 | 18,433799 |
| 11 | 19 | 443 | N/A | Cape Town | -34,040158 | 18,443332 |
| 12 | 26 | 786 | Kendal Road | Cape Town | -34,038481 | 18,45313 |
| 13 | 28 | 855 | Tortelduif Street | Stellenbosch | -33,939288 | 18,834178 |
| 14 | 167 | 1351 | N/A | Cape Town | -34,062296 | 18,414393 |
| 15 | 15 | 351 | Sohland Avenue | Cape Town | -34,029437 | 18,438486 |
| 15 | 13 | 8 | Strand Street | Cape Town | -33,926301 | 18,417396 |
| 17 | 16 | 658 | Parish Road | Cape Town | -34,023718 | 18,433759 |
| 18 | 50 | 1313 | Faerie Garden | Cape Town | -34,050433 | 18,408339 |
| 18 | 10 | 0 | N/A | Cape Town | -34,026322 | 18,44552 |
| 20 | 58 | 1461 | Loret Avenue | Cape Town | -34,021807 | 18,424068 |
| 20 | 57 | 1443 | Simon van der Stel Freeway | Cape Town | -34,042904 | 18,443016 |
| 22 | 9 | 0 | Vredenburg | Stellenbosch | -33,951278 | 18,822245 |
| 23 | 8 | 0 | Ribbok Street | Cape Town | -33,877955 | 18,708214 |
| 24 | 13 | 756 | N/A | Cape Town | -34,057303 | 18,435991 |
| 25 | 24 | 1353 | Charnwood Close | Cape Town | -34,066096 | 18,422193 |
| 26 | 21 | 1356 | Chapman's Peak Drive | Cape Town | -34,042533 | 18,365502 |
| 27 | 13 | 868 | N/A | Stellenbosch | -33,946337 | 18,833133 |
| 28 | 12 | 855 | Simon van der Stel Freeway | Cape Town | -34,02053 | 18,459787 |
| 29 | 96 | 1855 | Flamingo | Stellenbosch | -33,942092 | 18,844838 |
| 30 | 92 | 1859 | Thorpe Close | Cape Town | -34,06361 | 18,411541 |
| 31 | 30 | 1687 | Simon van der Stel Freeway | Cape Town | -34,04892 | 18,447314 |
| 31 | 12 | 916 | N/A | Cape Town | -33,96072 | 18,478209 |
| 33 | 7 | 101 | Settlers Way | Cape Town | -33,948056 | 18,482778 |
| 34 | 17 | 1412 | Paradise View Road | Cape Town | -33,989181 | 18,466839 |
| 35 | 16 | 1394 | Church Street | Cape Town | -33,961642 | 18,470702 |
| 35 | 12 | 1029 | Doncaster Road | Cape Town | -33,995999 | 18,483939 |
| 37 | 12 | 1053 | N/A | Cape Town | -33,954038 | 18,472796 |
| 38 | 37 | 1824 | Fish Eagles Way | Cape Town | -34,061803 | 18,503847 |
| 39 | 17 | 1492 | Main Road | Cape Town | -34,111632 | 18,460001 |
| 40 | 11 | 1029 | Peffers Street | Cape Town | -33,988862 | 18,473316 |
| 40 | 12 | 1090 | Saint James Street | Cape Town | -33,940091 | 18,430592 |
| 42 | 8 | 748 | Retreat Road | Cape Town | -34,049424 | 18,485126 |
| 43 | 11 | 1088 | Van Reede | Stellenbosch | -33,9546 | 18,831208 |
| 44 | 59 | 2090 | Tafelberg Road | Cape Town | -33,95121 | 18,434604 |
| 45 | 22 | 1740 | Abelia Street | Cape Town | -34,060136 | 18,823345 |
| 46 | 16 | 1622 | Bowlers Avenue | Cape Town | -33,84009 | 18,643552 |
| 46 | 7 | 649 | Old Kendal Road | Cape Town | -34,033073 | 18,459344 |
| 48 | 10 | 1072 | Glenmore Crescent | Cape Town | -34,045121 | 18,816361 |
| 48 | 131 | 2335 | N/A | Cape Town | -34,062207 | 18,399358 |
| 50 | 30 | 2090 | Tafelberg Road | Cape Town | -33,949478 | 18,431723 |
| 51 | 8 | 957 | Meyboom Avenue | Cape Town | -33,868282 | 18,579824 |
| 52 | 11 | 1397 | Country Side Road | Cape Town | -34,039414 | 18,37808 |
| 52 | 6 | 643 | Nursery Way | Cape Town | -33,937655 | 18,511095 |
| 54 | 11 | 1422 | Mount Prospect Drive | Cape Town | -34,037991 | 18,429115 |
| 55 | 4 | 0 | Pluimbos Close | Cape Town | -33,88071 | 18,71171 |
| 56 | 10 | 1368 | Settlers Way | Cape Town | -33,937573 | 18,476476 |
| 57 | 15 | 1740 | Main Road | Cape Town | -34,021216 | 18,46019 |
| 58 | 7 | 914 | N/A | Cape Town | -33,965294 | 18,495136 |
| 59 | 23 | 2061 | Southern Cross Drive | Cape Town | -34,01439 | 18,42655 |
| 60 | 8 | 1140 | Tarentaal | Stellenbosch | -33,936428 | 18,83155 |
| 61 | 13 | 1686 | N/A | Cape Town | -34,012835 | 18,455695 |
| 62 | 19 | 1968 | Edinburgh Drive | Cape Town | -34,003039 | 18,451674 |
| 62 | 16 | 1868 | Saffraan | Stellenbosch | -33,949298 | 18,838566 |
| 64 | 29 | 2321 | Bridle Road | Cape Town | -33,94688 | 18,423424 |
| 64 | 5 | 564 | Halyard Walk | Cape Town | -34,089833 | 18,472961 |
| 66 | 56 | 2581 | Tanja Road | Cape Town | -34,006904 | 18,438363 |
| 67 | 7 | 1038 | Macassar Road | Cape Town | -34,067344 | 18,758406 |
| 68 | 24 | 2301 | Childrens Way | Cape Town | -34,053433 | 18,456366 |
| 68 | 14 | 1788 | N/A | Stellenbosch | -34,039281 | 18,806989 |
| 70 | 16 | 1954 | Camps Bay Drive | Cape Town | -33,940187 | 18,394425 |
| 71 | 26 | 2356 | Totius Street | Cape Town | -33,87929 | 18,596619 |
| 72 | 5 | 693 | Valley Road | Cape Town | -34,01923 | 18,353419 |
| 73 | 126 | 2855 | Culemborg | Stellenbosch | -33,9432 | 18,848378 |
| 73 | 51 | 2662 | Level 5 | Cape Town | -34,07 | 18,4 |
| 75 | 4 | 319 | Spine Road | Cape Town | -34,017758 | 18,692942 |
| 76 | 15 | 1915 | Sandown Road | Cape Town | -33,973767 | 18,479003 |
| 77 | 5 | 756 | Lynx Close | Cape Town | -34,095896 | 18,452569 |
| 78 | 4 | 492 | Atlantic Road | Cape Town | -34,104415 | 18,464495 |
| 79 | 4 | 508 | Promenade Road | Cape Town | -34,090179 | 18,464602 |
| 80 | 6 | 1056 | Settlers Way | Cape Town | -33,94479 | 18,477604 |
| 80 | 5 | 798 | Woodgate Road | Cape Town | -34,016944 | 18,47165 |
| 82 | 3 | 0 | Voortrekker Road | Cape Town | -33,906792 | 18,580811 |
| 83 | 8 | 1484 | Ivy Lane | Cape Town | -34,064042 | 18,435514 |
| 84 | 8 | 1486 | Highlands Avenue | Cape Town | -33,942072 | 18,417271 |
| 85 | 35 | 2781 | N/A | Cape Town | -33,94926 | 18,454771 |
| 86 | 6 | 1145 | N/A | Stellenbosch | -33,936888 | 18,803987 |
| 87 | 8 | 1529 | Blue Valley Avenue | Cape Town | -34,020132 | 18,372733 |
| 87 | 17 | 2313 | Level 5 | Cape Town | -34,052768 | 18,405683 |
| 89 | 4 | 686 | Peak Drive | Cape Town | -33,942198 | 18,487884 |
| 89 | 42 | 2861 | N/A | Cape Town | -34,038616 | 18,399469 |
| 91 | 16 | 2313 | N/A | Cape Town | -33,947086 | 18,442539 |
| 92 | 45 | 2892 | Tanja Road | Cape Town | -34,007204 | 18,425663 |
| 93 | 8 | 1636 | Gilmour Hill Road | Cape Town | -33,923533 | 18,402097 |
| 94 | 5 | 1008 | Buitensingel Street | Cape Town | -33,924925 | 18,408384 |
| 94 | 4 | 754 | Devon Vallei | Stellenbosch | -33,933667 | 18,819613 |
| 94 | 66 | 3053 | North Lane | Cape Town | -33,954909 | 18,459407 |
| 97 | 12 | 2029 | Chichester Road | Cape Town | -33,982817 | 18,473406 |
| 97 | 36 | 2942 | Lovell | Stellenbosch | -33,957158 | 18,851162 |
| 99 | 10 | 1828 | Picketberg Way | Cape Town | -34,086404 | 18,443381 |
| 100 | 26 | 2807 | N/A | Cape Town | -33,973572 | 18,462819 |
| 101 | 14 | 2247 | Stanhope Road | Cape Town | -33,984038 | 18,467457 |
| 102 | 35 | 2988 | Price Drive | Cape Town | -34,026269 | 18,414947 |
| 102 | 55 | 3090 | Tafelberg Road | Cape Town | -33,95445 | 18,42695 |
| 104 | 7 | 1547 | Duinefontein Road | Cape Town | -34,001632 | 18,552648 |
| 105 | 118 | 3274 | N/A | Cape Town | -34,077872 | 18,40223 |
| 106 | 26 | 2866 | Saffraan | Stellenbosch | -33,947967 | 18,853792 |
| 107 | 4 | 911 | Bunker Road | Cape Town | -34,087864 | 18,457154 |
| 107 | 5 | 1215 | Government Avenue | Cape Town | -33,936337 | 18,40702 |
| 109 | 27 | 2953 | Merriman Avenue | Stellenbosch | -33,931747 | 18,851838 |
| 110 | 10 | 2003 | Bokmakierie | Stellenbosch | -33,93477 | 18,84483 |
| 111 | 5 | 1308 | Baden Powell Drive | Stellenbosch | -33,952629 | 18,80929 |
| 111 | 3 | 557 | N/A | Cape Town | -34,024449 | 18,443225 |
| 113 | 16 | 2663 | Zwaanswyk Road | Cape Town | -34,073491 | 18,407439 |
| 114 | 10 | 2089 | Baden Powell Drive | Stellenbosch | -33,971905 | 18,787663 |
| 115 | 7 | 1716 | Simon van der Stel Freeway | Cape Town | -34,055705 | 18,444078 |
| 116 | 18 | 2820 | Southern Cross Drive | Cape Town | -34,012353 | 18,418885 |
| 117 | 3 | 669 | N/A | Stellenbosch | -33,953575 | 18,817812 |
| 118 | 3 | 677 | Jutland Avenue | Cape Town | -33,930693 | 18,423462 |
| 119 | 3 | 683 | Firlands Meadow Road | Cape Town | -34,144036 | 18,894795 |
| 120 | 91 | 3473 | Southern Cross Drive | Cape Town | -34,007566 | 18,420059 |
| 121 | 58 | 3397 | Price Drive | Cape Town | -34,024414 | 18,413081 |
| 122 | 7 | 1756 | N/A | Cape Town | -34,099852 | 18,446053 |
| 123 | 4 | 1090 | N/A | Cape Town | -33,941882 | 18,435147 |
| 124 | 33 | 3316 | N/A | Cape Town | -34,082023 | 18,426288 |
| 125 | 4 | 1258 | George Starck Road | Cape Town | -33,894998 | 18,60925 |
| 125 | 3 | 782 | Valley Road | Cape Town | -34,020782 | 18,368264 |
| 127 | 16 | 2865 | Simon van der Stel Freeway | Cape Town | -34,074722 | 18,449666 |
| 127 | 24 | 3187 | N/A | Cape Town | -33,963385 | 18,459228 |
| 129 | 8 | 2006 | Baden Powell Drive | Stellenbosch | -33,95955 | 18,809275 |
| 129 | 60 | 3565 | N/A | Cape Town | -34,064062 | 18,39497 |
| 131 | 15 | 2815 | Hofmeyr Street | Cape Town | -33,876558 | 18,599572 |
| 132 | 2 | 0 | Garden Street | Cape Town | -33,916084 | 18,567047 |
| 132 | 4 | 1307 | Main Road | Cape Town | -34,041992 | 18,360338 |
| 132 | 2 | 0 | Plein Street | Cape Town | -33,825706 | 18,648603 |
| 135 | 3 | 810 | Kromboom Parkway | Cape Town | -33,999444 | 18,488611 |
| 136 | 3 | 848 | N/A | Cape Town | -33,931085 | 18,385262 |
| 137 | 25 | 3251 | N/A | Cape Town | -33,955158 | 18,417121 |
| 138 | 9 | 2185 | Campground Road | Cape Town | -33,973348 | 18,476748 |
| 139 | 3 | 874 | Uitsig Crescent | Cape Town | -33,837667 | 18,668258 |
| 140 | 10 | 2412 | N/A | Cape Town | -34,040925 | 18,399819 |
| 141 | 3 | 902 | N/A | Cape Town | -34,104389 | 18,456929 |
| 142 | 17 | 3048 | Spes Bona Valley | Cape Town | -34,1156 | 18,442 |
| 143 | 39 | 3567 | Level 5 | Cape Town | -34,07855 | 18,41005 |
| 144 | 18 | 3160 | N/A | Stellenbosch | -34,009967 | 18,760547 |
| 145 | 7 | 1967 | 58th Avenue | Cape Town | -34,004166 | 18,455278 |
| 145 | 2 | 29 | Main Road | Cape Town | -33,996645 | 18,476487 |
| 145 | 10 | 2510 | Military Road | Cape Town | -34,075486 | 18,450988 |
| 148 | 11 | 2655 | N/A | Cape Town | -34,034054 | 18,327366 |
| 149 | 6 | 1823 | N/A | Cape Town | -34,101563 | 18,447946 |
| 150 | 2 | 183 | N/A | Cape Town | -33,930315 | 18,479155 |
| 151 | 15 | 3018 | Hunters Way | Cape Town | -34,010713 | 18,376758 |
| 152 | 5 | 1707 | De Waal Road | Cape Town | -34,040058 | 18,463345 |
| 152 | 7 | 2055 | Settlers Way | Cape Town | -33,950306 | 18,46227 |
| 154 | 81 | 3855 | Andringa Street | Stellenbosch | -33,936972 | 18,856483 |
| 154 | 2 | 244 | Windermere Row | Cape Town | -34,09483 | 18,476156 |
| 156 | 2 | 249 | N/A | Cape Town | -33,886357 | 18,568485 |
| 157 | 29 | 3574 | N/A | Cape Town | -34,00124 | 18,436548 |
| 157 | 3 | 1060 | N/A | Cape Town | -33,93645 | 18,394747 |
| 157 | 2 | 292 | N/A | Cape Town | -33,887027 | 18,551197 |
| 160 | 6 | 1888 | Country Side Road | Cape Town | -34,04282 | 18,376188 |
| 161 | 11 | 2756 | N/A | Cape Town | -34,093997 | 18,434742 |
| 162 | 3 | 1089 | N/A | Stellenbosch | -33,974261 | 18,783966 |
| 163 | 2 | 323 | Nelson Mandela Boulevard | Cape Town | -33,921279 | 18,424055 |
| 164 | 8 | 2351 | Ou Wingerd Road | Cape Town | -34,029969 | 18,417725 |
| 165 | 28 | 3611 | Paradise Road | Cape Town | -33,9801 | 18,4511 |
| 165 | 20 | 3377 | N/A | Cape Town | -34,039943 | 18,395872 |
| 167 | 52 | 3805 | Hohenhort Avenue | Cape Town | -33,997377 | 18,427198 |
| 168 | 7 | 2176 | Morkel Road | Cape Town | -34,058113 | 18,453409 |
| 169 | 9 | 2557 | James Barry Avenue | Cape Town | -34,009243 | 18,443154 |
| 170 | 2 | 483 | Sheikh Yusuf Road | Cape Town | -34,070092 | 18,74832 |
| 170 | 3 | 1206 | Spier Wine Farm | Stellenbosch | -33,974984 | 18,782531 |
| 172 | 4 | 1596 | Parish Road | Cape Town | -34,013808 | 18,433279 |
| 173 | 32 | 3756 | N/A | Cape Town | -34,097714 | 18,426319 |
| 174 | 3 | 1244 | Baden Powell Drive | Cape Town | -34,099384 | 18,482374 |
| 174 | 2 | 504 | N/A | Cape Town | -34,021418 | 18,567165 |
| 176 | 24 | 3583 | Longkloof Road | Cape Town | -34,010101 | 18,37957 |
| 177 | 2 | 507 | N/A | Cape Town | -33,895865 | 18,732464 |
| 178 | 24 | 3595 | Rose Street | Cape Town | -33,98332 | 18,448167 |
| 179 | 3 | 1286 | Algoa Crescent | Cape Town | -33,868654 | 18,558563 |
| 179 | 6 | 2029 | Belvedere Road | Cape Town | -33,98207 | 18,481001 |
| 181 | 2 | 546 | Protea Way | Cape Town | -33,818328 | 18,64873 |
| 182 | 120 | 4215 | N/A | Cape Town | -34,009253 | 18,413677 |
| 183 | 4 | 1681 | First Avenue | Cape Town | -33,979167 | 18,496944 |
| 183 | 2 | 615 | Lichtenburg Road | Cape Town | -33,759004 | 18,766723 |
| 185 | 2 | 636 | Avenue Fresnaye | Cape Town | -33,927661 | 18,395355 |
| 185 | 22 | 3603 | Union Avenue | Cape Town | -33,970518 | 18,452171 |
| 187 | 3 | 1351 | Groot Constantia Road | Cape Town | -34,03114 | 18,428791 |
| 188 | 7 | 2335 | Chapman's Peak Drive | Cape Town | -34,054815 | 18,364869 |
| 189 | 8 | 2589 | Blackhill River Walk | Cape Town | -34,148985 | 18,394375 |
| 190 | 2 | 656 | N/A | Stellenbosch | -33,952962 | 18,827763 |
| 191 | 13 | 3179 | Campground Road | Cape Town | -33,975051 | 18,462234 |
| 192 | 4 | 1713 | Klein Constantia Road | Cape Town | -34,034815 | 18,416043 |
| 192 | 3 | 1370 | N/A | Stellenbosch | -33,934303 | 18,801887 |
| 194 | 3 | 1385 | Carrol Road | Cape Town | -34,022387 | 18,497667 |
| 194 | 2 | 684 | Nova Constantia Road | Cape Town | -34,041389 | 18,431111 |
| 194 | 82 | 4263 | N/A | Cape Town | -33,998415 | 18,416298 |
| 197 | 15 | 3363 | N/A | Cape Town | -34,03191 | 18,393219 |
| 198 | 3 | 1430 | Protea Way | Cape Town | -33,814844 | 18,659921 |
| 199 | 3 | 1469 | Rand Street | Cape Town | -33,81036 | 18,654033 |
| 200 | 28 | 3924 | Barry | Stellenbosch | -33,953181 | 18,856819 |
| 201 | 2 | 767 | Devon Vallei | Stellenbosch | -33,929263 | 18,818808 |
| 202 | 65 | 4328 | N/A | Cape Town | -34,0907 | 18,419 |
| 203 | 13 | 3302 | N/A | Cape Town | -34,054462 | 18,386957 |
| 204 | 2 | 781 | N/A | Cape Town | -33,93312 | 18,451467 |
| 205 | 2 | 782 | Groenewald Road | Cape Town | -34,026146 | 18,486793 |
| 206 | 3 | 1512 | Rabia Park Street | Cape Town | -33,954333 | 18,615618 |
| 207 | 80 | 4482 | Union Avenue | Cape Town | -33,969441 | 18,449208 |
| 208 | 3 | 1522 | Prince George Drive | Cape Town | -34,024139 | 18,479404 |
| 209 | 36 | 4240 | Union Avenue | Cape Town | -33,975494 | 18,442668 |
| 210 | 25 | 3928 | Merriman Avenue | Stellenbosch | -33,934236 | 18,862141 |
| 211 | 4 | 1843 | Kloof Street | Cape Town | -33,931571 | 18,397251 |
| 212 | 3 | 1557 | Constantia Main Road | Cape Town | -34,012573 | 18,449266 |
| 213 | 2 | 909 | Nelson Mandela Boulevard | Cape Town | -33,934413 | 18,450333 |
| 213 | 20 | 3848 | N/A | Stellenbosch | -34,01485 | 18,760497 |
| 213 | 12 | 3329 | N/A | Cape Town | -34,0606 | 18,3952 |
| 216 | 6 | 2497 | Strand Road | Stellenbosch | -33,962537 | 18,845911 |
| 217 | 51 | 4456 | Rose Street | Cape Town | -33,985278 | 18,44158 |
| 218 | 3 | 1643 | N/A | Cape Town | -33,918767 | 18,402753 |
| 219 | 2 | 932 | N/A | Cape Town | -33,826534 | 18,647362 |
| 220 | 2 | 942 | Nooitgedacht Drive | Cape Town | -34,018762 | 18,34757 |
| 221 | 9 | 3025 | De Villiers Avenue | Cape Town | -33,851844 | 18,633892 |
| 222 | 3 | 1672 | N/A | Cape Town | -34,109563 | 18,454643 |
| 223 | 4 | 1957 | Belvedere Avenue | Cape Town | -33,937001 | 18,413559 |
| 223 | 3 | 1679 | Gie Road | Cape Town | -33,814982 | 18,51682 |
| 223 | 2 | 968 | Waterloo Road | Cape Town | -33,998024 | 18,466829 |
| 226 | 2 | 988 | Main Road | Cape Town | -33,839554 | 18,643923 |
| 227 | 12 | 3455 | N/A | Cape Town | -33,89129 | 18,627304 |
| 228 | 9 | 3090 | N/A | Cape Town | -33,954197 | 18,439917 |
| 229 | 8 | 2968 | Simon van der Stel Freeway | Cape Town | -34,001687 | 18,44922 |
| 230 | 6 | 2637 | Rocket Road | Cape Town | -34,033031 | 18,331263 |
| 230 | 16 | 3790 | N/A | Cape Town | -34,086453 | 18,424056 |
| 232 | 2 | 1037 | Manatoka Avenue | Cape Town | -33,928412 | 18,689815 |
| 232 | 20 | 4043 | N/A | Cape Town | -34,085445 | 18,405408 |
| 234 | 4 | 2053 | N/A | Cape Town | -33,958012 | 18,464672 |
| 235 | 2 | 1059 | Yusuf Drive | Cape Town | -33,921362 | 18,407567 |
| 236 | 3 | 1729 | N/A | Stellenbosch | -33,8786 | 18,7647 |
| 236 | 10 | 3284 | N/A | Cape Town | -33,94902 | 18,40497 |
| 238 | 8 | 3029 | Campground Road | Cape Town | -33,975887 | 18,46972 |
| 239 | 21 | 4155 | Contour Path | Cape Town | -33,961739 | 18,446085 |
| 239 | 2 | 1074 | Robert Sobukwe Road | Cape Town | -33,942642 | 18,608831 |
| 239 | 28 | 4435 | Van Riebeeckshof Road | Cape Town | -33,859998 | 18,626978 |
| 239 | 18 | 4043 | N/A | Cape Town | -34,074675 | 18,395772 |
| 243 | 7 | 2861 | Marmion Road | Cape Town | -33,950899 | 18,410482 |
| 244 | 62 | 4753 | N/A | Cape Town | -33,989598 | 18,42829 |
| 245 | 4 | 2139 | Simon van der Stel Freeway | Cape Town | -34,064682 | 18,443233 |
| 246 | 10 | 3351 | Adam Tas Street | Stellenbosch | -33,927813 | 18,850595 |
| 247 | 6 | 2727 | Waveren Avenue | Cape Town | -34,051564 | 18,835712 |
| 248 | 8 | 3134 | N/A | Cape Town | -34,111555 | 18,434695 |
| 248 | 9 | 3245 | N/A | Cape Town | -33,954088 | 18,450887 |
| 250 | 7 | 2915 | Stanford Road | Cape Town | -33,974388 | 18,478762 |
| 251 | 1 | 0 | 5th Avenue | Cape Town | -33,89681 | 18,58302 |
| 251 | 1 | 0 | Bay View Heights Road | Cape Town | -34,180624 | 18,421313 |
| 251 | 1 | 0 | Giel Basson Drive | Cape Town | -33,913153 | 18,572538 |
| 251 | 1 | 0 | Kromboom Parkway | Cape Town | -33,956782 | 18,494133 |
| 251 | 1 | 0 | Macassar Road | Cape Town | -34,061313 | 18,751842 |
| 251 | 1 | 0 | Nelson Mandela Boulevard | Cape Town | -33,924869 | 18,424055 |
| 251 | 1 | 0 | Nelson Mandela Boulevard | Cape Town | -33,925148 | 18,433725 |
| 251 | 1 | 0 | Penguin Drive | Cape Town | -34,174136 | 18,427008 |
| 251 | 1 | 0 | Steynsrust Road | Cape Town | -34,05093 | 18,80623 |
| 251 | 1 | 0 | Wildevoelvlei Road | Cape Town | -34,136589 | 18,361597 |
| 251 | 1 | 0 | N/A | Cape Town | -34,02581 | 18,551112 |
| 251 | 1 | 0 | N/A | Cape Town | -33,832304 | 18,736165 |
| 263 | 15 | 3861 | Red Hill Road | Cape Town | -34,208938 | 18,401856 |
| 264 | 4 | 2220 | Edinburgh Drive | Cape Town | -33,993875 | 18,455466 |
| 265 | 15 | 3863 | Ackermann | Stellenbosch | -33,94517 | 18,860705 |
| 265 | 4 | 2230 | Polkadraai Road | Stellenbosch | -33,957066 | 18,799126 |
| 265 | 10 | 3434 | N/A | Cape Town | -34,024834 | 18,395412 |
| 268 | 6 | 2799 | N/A | Cape Town | -34,101638 | 18,435518 |
| 269 | 68 | 4887 | N/A | Cape Town | -34,0016 | 18,4115 |
| 270 | 4 | 2247 | Ring Road | Cape Town | -33,961231 | 18,460403 |
| 271 | 13 | 3765 | Hout Bay Main Road | Cape Town | -34,014079 | 18,391026 |
| 272 | 18 | 4236 | Jan Cilliers | Stellenbosch | -33,92717 | 18,857922 |
| 273 | 5 | 2604 | N/A | Cape Town | -34,115558 | 18,458211 |
| 274 | 2 | 1316 | Steenberg Road | Cape Town | -34,066112 | 18,424823 |
| 275 | 5 | 2643 | N/A | Cape Town | -34,153928 | 18,38361 |
| 275 | 3 | 1848 | N/A | Cape Town | -33,942256 | 18,380812 |
| 277 | 4 | 2309 | N/A | Cape Town | -34,195 | 18,403309 |
| 278 | 5 | 2649 | Camilla Street | Cape Town | -34,151941 | 18,42536 |
| 279 | 43 | 4855 | Pastorie | Stellenbosch | -33,942683 | 18,872703 |
| 280 | 11 | 3670 | Broadway Boulevard | Cape Town | -34,089105 | 18,813159 |
| 281 | 3 | 1898 | Zandberg Street | Cape Town | -34,046476 | 18,823099 |
| 282 | 3 | 1910 | Murray Street | Cape Town | -33,807122 | 18,657836 |
| 283 | 2 | 1371 | Park Road | Cape Town | -34,013248 | 18,460554 |
| 284 | 18 | 4350 | N/A | Cape Town | -34,022421 | 18,405836 |
| 285 | 2 | 1393 | N/A | Cape Town | -34,195433 | 18,415961 |
| 285 | 1 | 165 | N/A | Cape Town | -33,760254 | 18,775249 |
| 287 | 1 | 174 | N/A | Stellenbosch | -33,833599 | 18,794586 |
| 288 | 1 | 176 | Fourth Avenue | Cape Town | -34,058538 | 18,469836 |
| 288 | 5 | 2720 | M. Tohalisi Street | Cape Town | -33,99641 | 18,670682 |
| 290 | 3 | 1940 | N/A | Cape Town | -34,169459 | 18,399317 |
| 291 | 1 | 192 | Rusthof Street | Cape Town | -34,134033 | 18,842539 |
| 291 | 3 | 1950 | Vakwana Street | Cape Town | -34,031044 | 18,672295 |
| 293 | 1 | 210 | Northpine Drive | Cape Town | -33,871406 | 18,711515 |
| 293 | 4 | 2453 | Sterling Street | Cape Town | -33,807496 | 18,657551 |
| 295 | 1 | 223 | Blaauwberg Road | Cape Town | -33,835234 | 18,517665 |
| 296 | 2 | 1457 | N/A | Cape Town | -33,993145 | 18,492079 |
| 297 | 112 | 5366 | N/A | Cape Town | -33,987057 | 18,431785 |
| 297 | 1 | 298 | N/A | Cape Town | -33,758746 | 18,784914 |
| 299 | 36 | 4914 | Hospitaal | Stellenbosch | -33,931274 | 18,867897 |
| 299 | 1 | 307 | Main Road | Cape Town | -34,039965 | 18,358162 |
| 301 | 8 | 3447 | Mousebird Way | Cape Town | -34,072177 | 18,833797 |
| 301 | 1 | 323 | Nelson Mandela Boulevard | Cape Town | -33,914638 | 18,416655 |
| 301 | 10 | 3715 | N/A | Cape Town | -34,1207 | 18,441144 |
| 304 | 11 | 3807 | Beachcomber Bay | Cape Town | -34,141417 | 18,327722 |
| 304 | 1 | 392 | N/A | Cape Town | -33,951612 | 18,520537 |
| 306 | 1 | 393 | Neptune Close | Cape Town | -34,184154 | 18,423087 |
| 306 | 4 | 2562 | N/A | Stellenbosch | -33,972755 | 18,80775 |
| 308 | 30 | 4912 | Nooitgedacht | Stellenbosch | -33,954675 | 18,86648 |
| 308 | 1 | 418 | Vasco Boulevard | Cape Town | -33,911672 | 18,552253 |
| 310 | 13 | 4106 | Broadway Boulevard | Stellenbosch | -33,97573 | 18,845013 |
| 311 | 1 | 475 | Sunbush Close | Cape Town | -34,038683 | 18,36977 |
| 312 | 3 | 2092 | Frans Conradie Drive | Cape Town | -33,894987 | 18,61832 |
| 313 | 1 | 483 | Annandale Road | Stellenbosch | -33,990653 | 18,800588 |
| 314 | 7 | 3312 | Strand Road | Stellenbosch | -33,961125 | 18,855167 |
| 315 | 34 | 5047 | Hout Bay Main Road | Cape Town | -34,008611 | 18,40579 |
| 315 | 1 | 499 | President Swart Street | Cape Town | -33,883678 | 18,573218 |
| 315 | 37 | 5127 | N/A | Cape Town | -33,993378 | 18,41567 |
| 318 | 13 | 4208 | Coronata | Stellenbosch | -33,963898 | 18,861276 |
| 318 | 8 | 3543 | N/A | Cape Town | -34,030572 | 18,404412 |
| 318 | 1 | 506 | N/A | Cape Town | -34,024843 | 18,691807 |
| 321 | 20 | 4727 | Alston Lane | Cape Town | -34,051178 | 18,852858 |
| 322 | 2 | 1583 | Bergvliet Road | Cape Town | -34,047938 | 18,454311 |
| 322 | 3 | 2174 | Blue Valley Avenue | Cape Town | -34,015485 | 18,372086 |
| 322 | 12 | 4099 | Rhodes Avenue | Cape Town | -33,989722 | 18,435556 |
| 322 | 1 | 526 | N/A | Cape Town | -33,785897 | 18,508181 |
| 326 | 1 | 549 | Beach Road | Cape Town | -34,104114 | 18,470723 |
| 327 | 2 | 1608 | Howard Drive | Cape Town | -33,932396 | 18,515807 |
| 328 | 1 | 567 | Sir Lowry's Pass Road | Cape Town | -34,150513 | 18,877974 |
| 328 | 28 | 5044 | Van Riebeeckshof Road | Cape Town | -33,857707 | 18,616683 |
| 330 | 1 | 594 | Ross Street | Cape Town | -33,958807 | 18,572159 |
| 330 | 4 | 2703 | Stanley Place | Cape Town | -33,903358 | 18,401792 |
| 330 | 16 | 4621 | N/A | Cape Town | -34,011511 | 18,40575 |
| 333 | 2 | 1649 | Glass Furnace Way | Cape Town | -34,157086 | 18,428419 |
| 333 | 16 | 4647 | Webersvallei Road | Stellenbosch | -33,980892 | 18,853833 |
| 335 | 3 | 2271 | N/A | Cape Town | -33,986534 | 18,49321 |
| 336 | 1 | 649 | Glen Road | Cape Town | -34,161847 | 18,429907 |
| 336 | 3 | 2273 | N/A | Stellenbosch | -33,933303 | 18,800708 |
| 338 | 2 | 1680 | Flight Road | Cape Town | -34,050802 | 18,509713 |
| 339 | 15 | 4557 | N/A | Cape Town | -33,95971 | 18,412913 |
| 340 | 24 | 4991 | Birch Street | Cape Town | -33,9777 | 18,4351 |
| 341 | 1 | 710 | Settlers Way | Cape Town | -33,94241 | 18,479675 |
| 342 | 1 | 722 | Cardiff Street | Cape Town | -33,813789 | 18,69464 |
| 343 | 3 | 2344 | Fivaz Street | Cape Town | -33,843634 | 18,637919 |
| 343 | 1 | 727 | Sauvignon Street | Cape Town | -34,052166 | 18,81505 |
| 345 | 1 | 737 | Sir Lowry's Pass Road | Cape Town | -34,158639 | 18,87462 |
| 346 | 6 | 3327 | Chapman's Peak Drive | Cape Town | -34,056811 | 18,366597 |
| 347 | 1 | 752 | Klein Constantia Road | Cape Town | -34,0462 | 18,419849 |
| 348 | 2 | 1732 | Ruyteplaats Drive | Cape Town | -34,015826 | 18,361255 |
| 349 | 10 | 4090 | Contour Path | Cape Town | -33,96269 | 18,435938 |
| 349 | 18 | 4862 | Park | Stellenbosch | -33,95008 | 18,86563 |
| 349 | 2 | 1735 | Princess Vlei Road | Cape Town | -34,02783 | 18,474156 |
| 352 | 1 | 757 | Acacia Street | Cape Town | -34,058698 | 18,817972 |
| 353 | 1 | 766 | Prince George Drive | Cape Town | -34,015801 | 18,481772 |
| 354 | 24 | 5142 | Trumali Street | Stellenbosch | -33,965187 | 18,866772 |
| 354 | 19 | 4897 | N/A | Cape Town | -34,08762 | 18,40379 |
| 354 | 2 | 1744 | N/A | Cape Town | -34,074443 | 18,760106 |
| 357 | 1 | 769 | Chatham Road | Cape Town | -34,051819 | 18,469942 |
| 358 | 1 | 774 | Baden Powell Drive | Cape Town | -34,096492 | 18,4904 |
| 358 | 3 | 2468 | Leeuwenhof Crescent | Cape Town | -33,942021 | 18,405125 |
| 360 | 8 | 3788 | N/A | Cape Town | -34,103611 | 18,429138 |
| 361 | 2 | 1780 | Victoria Road | Cape Town | -34,0188 | 18,470367 |
| 362 | 1 | 791 | Settlers Way | Cape Town | -33,94402 | 18,524862 |
| 363 | 1 | 791 | Rameron Avenue | Cape Town | -34,137203 | 18,359705 |
| 364 | 1 | 809 | Ennerdale | Cape Town | -33,928939 | 18,508772 |
| 364 | 15 | 4756 | Ou Kaapse Weg | Cape Town | -34,092961 | 18,422425 |
| 366 | 1 | 842 | N/A | Cape Town | -34,181381 | 18,407233 |
| 367 | 12 | 4510 | N/A | Cape Town | -34,068159 | 18,386621 |
| 368 | 12 | 4512 | Old Wagon Road | Cape Town | -34,08573 | 18,409092 |
| 368 | 2 | 1812 | N/A | Cape Town | -34,147227 | 18,381365 |
| 368 | 14 | 4688 | N/A | Cape Town | -34,0787 | 18,3933 |
| 371 | 46 | 5855 | Mancadan | Stellenbosch | -33,937992 | 18,879322 |
| 371 | 3 | 2568 | N/A | Cape Town | -34,195684 | 18,44943 |
| 373 | 2 | 1820 | Troon Crescent | Cape Town | -33,798889 | 18,482759 |
| 373 | 11 | 4433 | N/A | Cape Town | -34,149192 | 18,928131 |
| 375 | 3 | 2582 | Viridian Drive | Cape Town | -33,839077 | 18,553915 |
| 375 | 6 | 3501 | W. R. Quinan Boulevard | Cape Town | -34,087524 | 18,809721 |
| 377 | 1 | 877 | Sydow Street | Cape Town | -33,862619 | 18,733675 |
| 378 | 1 | 891 | Vatican Street | Cape Town | -33,81542 | 18,690387 |
| 379 | 2 | 1840 | N/A | Stellenbosch | -33,97922 | 18,805817 |
| 380 | 1 | 916 | Kayla Close | Cape Town | -33,800021 | 18,489106 |
| 381 | 2 | 1870 | Techno Avenue | Stellenbosch | -33,965583 | 18,833047 |
| 382 | 1 | 946 | Albert Road | Cape Town | -33,935189 | 18,463506 |
| 383 | 1 | 946 | Breitenbach Street | Cape Town | -33,9914 | 18,7275 |
| 383 | 26 | 5656 | Constantia Corner | Cape Town | -33,994389 | 18,41325 |
| 383 | 18 | 5162 | Hospitaal | Stellenbosch | -33,926415 | 18,872065 |
| 386 | 1 | 953 | Flintdale Road | Cape Town | -34,047603 | 18,482504 |
| 387 | 8 | 4064 | N/A | Cape Town | -34,062928 | 18,386159 |
| 388 | 3 | 2693 | Ruyteplaats Drive | Cape Town | -34,008674 | 18,360868 |
| 388 | 1 | 967 | Waterloo Road | Cape Town | -34,005734 | 18,466241 |
| 390 | 3 | 2699 | Baden Powell Drive | Stellenbosch | -33,961945 | 18,79963 |
| 391 | 2 | 1914 | Kromboom Road | Cape Town | -33,96881 | 18,491309 |
| 392 | 10 | 4448 | N/A | Cape Town | -34,124517 | 18,439949 |
| 393 | 31 | 5904 | Jannasch | Stellenbosch | -33,93535 | 18,876667 |
| 393 | 2 | 1930 | N/A | Cape Town | -34,03801 | 18,813461 |
| 395 | 1 | 1021 | Van Riebeeck Road | Cape Town | -33,925708 | 18,683217 |
| 396 | 4 | 3142 | N/A | Cape Town | -34,079787 | 18,436081 |
| 397 | 9 | 4308 | Hout Bay Main Road | Cape Town | -34,00785 | 18,390756 |
| 397 | 4 | 3180 | Zevendal Way | Cape Town | -33,942983 | 18,712202 |
| 399 | 1 | 1067 | Aandblom | Cape Town | -33,940703 | 18,504448 |
| 400 | 3 | 2791 | Southern Right Circle | Cape Town | -34,135728 | 18,334403 |
| 400 | 7 | 3908 | N/A | Cape Town | -34,124538 | 18,44809 |
| 402 | 15 | 5090 | Fernwood Track | Cape Town | -33,972496 | 18,438961 |
| 403 | 6 | 3747 | Kommissaris Street | Cape Town | -33,874827 | 18,605796 |
| 404 | 6 | 3755 | N/A | Cape Town | -34,180558 | 18,908222 |
| 405 | 28 | 6098 | Brandwacht | Stellenbosch | -33,960563 | 18,875295 |
| 406 | 1 | 1123 | Rose Crescent | Cape Town | -33,939979 | 18,628872 |
| 407 | 59 | 6855 | Du Plessis | Stellenbosch | -33,941675 | 18,886788 |
| 407 | 4 | 3247 | Strand Road | Stellenbosch | -33,969472 | 18,841787 |
| 409 | 2 | 2082 | Crane Crescent | Cape Town | -34,065997 | 18,528568 |
| 410 | 17 | 5458 | Plateau Street | Cape Town | -34,222409 | 18,408101 |
| 410 | 1 | 1149 | Plein Street | Cape Town | -33,817986 | 18,64435 |
| 412 | 1 | 1176 | Main Road | Cape Town | -34,061346 | 18,466029 |
| 413 | 12 | 4860 | Emerald Drive | Cape Town | -34,101725 | 18,405838 |
| 413 | 1 | 1185 | Monte Vista Boulevard | Cape Town | -33,871303 | 18,555795 |
| 415 | 6 | 3788 | Watson Way | Stellenbosch | -34,019178 | 18,807062 |
| 415 | 1 | 1202 | White Road | Cape Town | -34,063793 | 18,460798 |
| 417 | 2 | 2110 | Main Road | Cape Town | -34,067713 | 18,826123 |
| 417 | 1 | 1207 | N/A | Cape Town | -33,905123 | 18,510377 |
| 419 | 1 | 1212 | N/A | Cape Town | -34,068167 | 18,815416 |
| 420 | 2 | 2144 | Baden Powell Drive | Stellenbosch | -33,96765 | 18,801079 |
| 421 | 2 | 2150 | Beach Road | Cape Town | -34,164438 | 18,870842 |
| 422 | 1 | 1270 | Durmonte Drive | Cape Town | -33,81196 | 18,673065 |
| 422 | 8 | 4334 | Main Road | Cape Town | -34,074081 | 18,839621 |
| 422 | 22 | 5861 | N/A | Stellenbosch | -33,948958 | 18,877467 |
| 425 | 1 | 1277 | Heron Road | Cape Town | -34,060372 | 18,521154 |
| 426 | 3 | 2906 | N/A | Cape Town | -34,173676 | 18,391924 |
| 427 | 1 | 1299 | Baden Powell Drive | Cape Town | -34,074864 | 18,582405 |
| 428 | 2 | 2180 | N/A | Cape Town | -33,94288 | 18,710926 |
| 429 | 4 | 3331 | Ou Kaapse Weg | Cape Town | -34,08521 | 18,418543 |
| 430 | 3 | 2918 | Dal Road | Cape Town | -33,944318 | 18,39445 |
| 431 | 21 | 5905 | Brandwacht | Stellenbosch | -33,957645 | 18,876713 |
| 431 | 1 | 1316 | N/A | Cape Town | -33,959282 | 18,503983 |
| 433 | 1 | 1323 | Bascule Bridge | Cape Town | -33,914111 | 18,416636 |
| 433 | 5 | 3686 | N/A | Cape Town | -33,953189 | 18,413992 |
| 435 | 1 | 1327 | Valmar Street | Cape Town | -33,840896 | 18,651016 |
| 436 | 28 | 6730 | Upper Mountain Road | Cape Town | -34,060197 | 18,864482 |
| 437 | 1 | 1366 | N/A | Cape Town | -34,148164 | 18,377615 |
| 438 | 2 | 2302 | Kaag Crescent | Cape Town | -34,125607 | 18,394306 |
| 438 | 6 | 3967 | Masitandane Road | Stellenbosch | -33,921495 | 18,852587 |
| 438 | 3 | 3018 | Plettenberg Street | Cape Town | -33,877276 | 18,612516 |
| 441 | 14 | 5319 | N/A | Cape Town | -34,07723 | 18,366311 |
| 442 | 3 | 3020 | N/A | Cape Town | -34,199032 | 18,404701 |
| 443 | 1 | 1371 | N/A | Cape Town | -33,99848 | 18,622685 |
| 444 | 9 | 4692 | Cynaroides | Stellenbosch | -33,970475 | 18,856772 |
| 444 | 11 | 4974 | N/A | Cape Town | -34,111181 | 18,4095 |
| 446 | 3 | 3029 | Durban Road | Cape Town | -33,894643 | 18,622864 |
| 447 | 3 | 3054 | Brackenfell Boulevard | Cape Town | -33,860957 | 18,675541 |
| 447 | 4 | 3483 | Stellenrust Road | Stellenbosch | -33,994542 | 18,82741 |
| 449 | 11 | 4995 | N/A | Cape Town | -34,00087 | 18,3911 |
| 449 | 6 | 4090 | N/A | Cape Town | -33,959579 | 18,427034 |
| 451 | 21 | 6413 | N/A | Cape Town | -33,978212 | 18,41377 |
| 452 | 1 | 1449 | Edgemead Drive | Cape Town | -33,877525 | 18,536801 |
| 453 | 3 | 3113 | N/A | Cape Town | -34,080588 | 18,775862 |
| 454 | 1 | 1472 | N/A | Cape Town | -33,893991 | 18,722642 |
| 455 | 8 | 4655 | Village Lane | Cape Town | -34,097593 | 18,373655 |
| 455 | 1 | 1473 | N/A | Cape Town | -33,938593 | 18,446242 |
| 457 | 21 | 6471 | Erasmus Smit | Stellenbosch | -33,919005 | 18,881597 |
| 457 | 1 | 1481 | Klein Constantia Road | Cape Town | -34,043481 | 18,406319 |
| 457 | 5 | 3845 | Noordhoek Road | Cape Town | -34,10659 | 18,379638 |
| 460 | 1 | 1483 | N/A | Cape Town | -34,07163 | 18,755603 |
| 461 | 4 | 3577 | Chapman's Peak Drive | Cape Town | -34,05782 | 18,37687 |
| 461 | 14 | 5582 | N/A | Stellenbosch | -33,916742 | 18,868878 |
| 461 | 1 | 1485 | N/A | Cape Town | -34,075277 | 18,739444 |
| 461 | 13 | 5473 | N/A | Cape Town | -33,972403 | 18,414886 |
| 465 | 5 | 3862 | N/A | Cape Town | -34,188202 | 18,381203 |
| 466 | 1 | 1517 | Baden Powell Drive | Stellenbosch | -33,988733 | 18,765982 |
| 466 | 9 | 4848 | Ingleside Road | Cape Town | -33,963265 | 18,384165 |
| 468 | 1 | 1519 | N/A | Cape Town | -34,044062 | 18,744112 |
| 469 | 1 | 1519 | Baden Powell Drive | Stellenbosch | -33,983137 | 18,773542 |
| 470 | 1 | 1520 | Jakes Gerwel Drive | Cape Town | -33,986566 | 18,536007 |
| 470 | 3 | 3222 | Wavell Avenue | Cape Town | -34,198601 | 18,447101 |
| 472 | 5 | 3919 | Cyprus Road | Cape Town | -34,063734 | 18,844689 |
| 473 | 1 | 1528 | Ivory Close | Cape Town | -34,081863 | 18,473755 |
| 474 | 1 | 1533 | Skilpaddam Road | Cape Town | -33,946838 | 18,700799 |
| 475 | 30 | 7695 | Radloff Park Through | Cape Town | -34,081009 | 18,875168 |
| 476 | 3 | 3256 | Kuils River Freeway | Cape Town | -33,879044 | 18,671415 |
| 477 | 3 | 3256 | N/A | Cape Town | -33,875855 | 18,670275 |
| 477 | 1 | 1566 | N/A | Cape Town | -33,804825 | 18,513558 |
| 479 | 1 | 1577 | Ryger Street | Cape Town | -34,162157 | 18,875956 |
| 479 | 20 | 6793 | N/A | Cape Town | -33,973889 | 18,4 |
| 479 | 15 | 5913 | N/A | Cape Town | -33,973401 | 18,399383 |
| 482 | 1 | 1587 | Vygeboom Road | Cape Town | -33,843783 | 18,668022 |
| 483 | 15 | 6034 | Contour Path | Cape Town | -33,985012 | 18,419146 |
| 484 | 1 | 1621 | Morningside Road | Cape Town | -33,936264 | 18,512636 |
| 484 | 17 | 6489 | N/A | Cape Town | -33,982334 | 18,413813 |
| 486 | 14 | 5818 | Contour Path | Cape Town | -33,980853 | 18,426651 |
| 486 | 5 | 4087 | N/A | Cape Town | -34,195479 | 18,383039 |
| 488 | 1 | 1649 | Main Road | Cape Town | -34,031265 | 18,46047 |
| 489 | 5 | 4100 | Van Riebeeckshof Road | Cape Town | -33,855407 | 18,609042 |
| 490 | 2 | 2658 | Katzenellenbogen Road | Cape Town | -34,110401 | 18,37752 |
| 490 | 15 | 6173 | Skeleton Gorge | Cape Town | -33,979619 | 18,415771 |
| 490 | 1 | 1661 | N/A | Stellenbosch | -33,963207 | 18,81224 |
| 493 | 19 | 6877 | Three Firs | Cape Town | -33,973889 | 18,396111 |
| 493 | 1 | 1663 | Zandkloof Road | Cape Town | -33,831837 | 18,680878 |
| 493 | 2 | 2659 | N/A | Stellenbosch | -33,970421 | 18,810326 |
| 496 | 1 | 1663 | Main Road | Cape Town | -33,912457 | 18,407637 |
| 497 | 1 | 1676 | Daniels Crescent | Cape Town | -34,052825 | 18,501119 |
| 497 | 26 | 7730 | Verster Avenue | Cape Town | -34,05738 | 18,87441 |
| 499 | 2 | 2718 | Ruyteplaats Drive | Cape Town | -34,008816 | 18,364702 |
| 500 | 5 | 4213 | Platteklip Gorge | Cape Town | -33,959316 | 18,416368 |
| 500 | 1 | 1693 | Ruyteplaats Drive | Cape Town | -34,018336 | 18,359187 |
| 502 | 1 | 1696 | Dunedin Road | Cape Town | -34,154969 | 18,417583 |
| 503 | 2 | 2735 | Saint Emillion Road | Cape Town | -34,057819 | 18,828552 |
| 503 | 1 | 1699 | Seaton Street | Cape Town | -34,03449 | 18,469389 |
| 503 | 5 | 4230 | N/A | Cape Town | -34,171727 | 18,919046 |
| 506 | 4 | 3848 | Central Drive | Cape Town | -33,958572 | 18,383358 |
| 506 | 2 | 2740 | Ruby Road | Cape Town | -33,9821 | 18,675275 |
| 508 | 7 | 4763 | N/A | Cape Town | -34,216358 | 18,398781 |
| 509 | 1 | 1727 | Beaulie Crescent | Cape Town | -34,051035 | 18,824235 |
| 509 | 7 | 4804 | Kelp Road | Cape Town | -34,138749 | 18,324624 |
| 509 | 2 | 2757 | Ou Kaapse Weg | Cape Town | -34,118101 | 18,394521 |
| 512 | 3 | 3468 | N/A | Cape Town | -34,172595 | 18,918016 |
| 513 | 1 | 1737 | Creekside Road | Cape Town | -33,789347 | 18,485758 |
| 514 | 2 | 2805 | Century Avenue | Cape Town | -33,886125 | 18,51763 |
| 515 | 1 | 1749 | Dyke Road | Cape Town | -33,994856 | 18,734775 |
| 516 | 1 | 1757 | Dover Road | Cape Town | -34,041439 | 18,476027 |
| 517 | 1 | 1758 | Cedarwood Street | Cape Town | -33,833061 | 18,682158 |
| 517 | 2 | 2826 | N/A | Cape Town | -33,982084 | 18,45146 |
| 519 | 1 | 1767 | Bamboesvlei Road | Cape Town | -34,004796 | 18,508788 |
| 520 | 7 | 4880 | Impala Road | Cape Town | -34,065678 | 18,847837 |
| 521 | 1 | 1781 | N/A | Cape Town | -33,941895 | 18,457711 |
| 522 | 4 | 4041 | N/A | Cape Town | -34,112866 | 18,432811 |
| 522 | 1 | 1782 | N/A | Cape Town | -34,073152 | 18,511868 |
| 524 | 17 | 7381 | Drama Street | Cape Town | -34,08248 | 18,871427 |
| 524 | 1 | 1798 | Remo Close | Cape Town | -33,892991 | 18,705747 |
| 526 | 1 | 1803 | Zonnekus Road | Cape Town | -33,754167 | 18,548611 |
| 527 | 1 | 1809 | Winery Road | Stellenbosch | -34,034153 | 18,79248 |
| 528 | 1 | 1809 | Ruyteplaats Drive | Cape Town | -34,013503 | 18,343971 |
| 529 | 2 | 2869 | Hoerikwaggo Trail | Cape Town | -34,185609 | 18,395077 |
| 530 | 1 | 1815 | Keurboom Crescent | Cape Town | -33,870522 | 18,591108 |
| 530 | 10 | 5731 | Oldenland Road | Cape Town | -34,056162 | 18,864067 |
| 532 | 5 | 4483 | Stellenrust Road | Stellenbosch | -33,992217 | 18,831362 |
| 533 | 1 | 1828 | Burgundy Drive | Cape Town | -33,840934 | 18,544719 |
| 533 | 2 | 2913 | Noordhoek Road | Cape Town | -34,114257 | 18,379956 |
| 535 | 18 | 7821 | Verster Avenue | Cape Town | -34,066266 | 18,874757 |
| 536 | 2 | 2955 | Milky Way | Cape Town | -34,155309 | 18,344573 |
| 536 | 3 | 3693 | N/A | Cape Town | -34,001519 | 18,356177 |
| 538 | 3 | 3695 | Constantia Main Road | Cape Town | -34,013702 | 18,406501 |
| 538 | 5 | 4526 | Eland Street | Cape Town | -34,20141 | 18,382022 |
| 540 | 1 | 1883 | Serruria Close | Cape Town | -33,862702 | 18,593476 |
| 541 | 10 | 5882 | Kasteelspoort Path | Cape Town | -33,970627 | 18,396463 |
| 542 | 6 | 4860 | Steenbras Dam Road | Cape Town | -34,181525 | 18,847586 |
| 542 | 1 | 1901 | N/A | Cape Town | -33,909796 | 18,617223 |
| 544 | 15 | 7388 | Luckhoff | Stellenbosch | -33,918603 | 18,882433 |
| 545 | 1 | 1912 | Steenoven Street | Cape Town | -33,86009 | 18,552215 |
| 546 | 5 | 4647 | N/A | Cape Town | -34,038242 | 18,309177 |
| 547 | 8 | 5546 | Paradyskloof Road | Stellenbosch | -33,970238 | 18,872095 |
| 548 | 1 | 1932 | Vissershokpad | Cape Town | -33,82979 | 18,633391 |
| 549 | 33 | 9890 | Jonkershoek Road | Stellenbosch | -33,958192 | 18,913617 |
| 549 | 8 | 5571 | Old Wagon Road | Cape Town | -34,107 | 18,4095 |
| 549 | 1 | 1934 | Ottery Road | Cape Town | -34,018097 | 18,503376 |
| 552 | 4 | 4317 | Steenbras Dam Road | Cape Town | -34,175627 | 18,859537 |
| 553 | 1 | 1959 | Aurora Street | Cape Town | -33,838842 | 18,633961 |
| 554 | 2 | 3090 | N/A | Cape Town | -33,853099 | 18,586188 |
| 555 | 10 | 6236 | Koelenhof Road | Stellenbosch | -33,887428 | 18,828213 |
| 555 | 16 | 7892 | Omega | Stellenbosch | -33,931137 | 18,892142 |
| 555 | 13 | 7081 | Van Der Merwe Road | Cape Town | -34,073242 | 18,869439 |
| 555 | 7 | 5255 | N/A | Cape Town | -33,990801 | 18,387893 |
| 559 | 3 | 3798 | Watson Way | Stellenbosch | -34,025517 | 18,798113 |
| 559 | 1 | 1987 | N/A | Cape Town | -34,055295 | 18,768039 |
| 561 | 4 | 4384 | Outer Orange Kloof Ring Road | Cape Town | -34,003515 | 18,384397 |
| 561 | 1 | 2022 | N/A | Cape Town | -33,921623 | 18,624524 |
| 563 | 4 | 4426 | Red Hill Road | Cape Town | -34,211833 | 18,395145 |
| 564 | 2 | 3203 | Howard Davis Road | Cape Town | -33,89016 | 18,513193 |
| 564 | 1 | 2085 | N/A | Cape Town | -33,887008 | 18,605873 |
| 566 | 10 | 6612 | Disa Gorge | Cape Town | -33,982584 | 18,39077 |
| 566 | 8 | 5854 | Reservoir Road | Cape Town | -34,063947 | 18,864034 |
| 568 | 4 | 4517 | N/A | Stellenbosch | -34,021341 | 18,753749 |
| 568 | 5 | 4858 | N/A | Cape Town | -34,186577 | 18,376 |
| 570 | 1 | 2161 | Wood Way | Cape Town | -34,1588 | 18,412654 |
| 570 | 12 | 7364 | N/A | Stellenbosch | -33,97268 | 18,885387 |
| 572 | 1 | 2169 | N/A | Cape Town | -34,077813 | 18,515597 |
| 573 | 1 | 2171 | Parklands Main Road | Cape Town | -33,814808 | 18,50581 |
| 573 | 3 | 4021 | N/A | Cape Town | -34,210553 | 18,446004 |
| 575 | 7 | 5620 | Outer Orange Kloof Ring Road | Cape Town | -34,000976 | 18,400685 |
| 576 | 4 | 4610 | N/A | Stellenbosch | -34,018683 | 18,758175 |
| 577 | 7 | 5650 | Old Wagon Road | Cape Town | -34,096184 | 18,40589 |
| 578 | 1 | 2236 | Java Close | Cape Town | -34,137985 | 18,390291 |
| 579 | 1 | 2241 | Almond Street | Cape Town | -33,86812 | 18,544095 |
| 579 | 5 | 4896 | N/A | Cape Town | -34,075289 | 18,384326 |
| 581 | 2 | 3319 | N/A | Cape Town | -34,015546 | 18,657566 |
| 582 | 2 | 3328 | Anneke Road | Cape Town | -33,856412 | 18,663642 |
| 582 | 1 | 2268 | Beethoven Crescent | Cape Town | -33,840111 | 18,682387 |
| 582 | 8 | 6137 | N/A | Stellenbosch | -33,910533 | 18,871597 |
| 585 | 3 | 4140 | N/A | Cape Town | -33,8986 | 18,6489 |
| 586 | 1 | 2309 | Duinefontein Road | Cape Town | -33,981203 | 18,554127 |
| 586 | 10 | 6960 | Woodie's Walk | Cape Town | -34,03947 | 18,864386 |
| 588 | 2 | 3383 | N/A | Cape Town | -34,041997 | 18,396834 |
| 589 | 1 | 2316 | Steenberg Road | Cape Town | -34,076875 | 18,428717 |
| 590 | 1 | 2328 | Hebron Street | Cape Town | -33,849794 | 18,661754 |
| 590 | 2 | 3424 | N/A | Cape Town | -33,867357 | 18,666992 |
| 592 | 2 | 3450 | Silvermine Road | Cape Town | -34,110112 | 18,393608 |
| 592 | 11 | 7727 | Watsonia Trail | Cape Town | -34,055057 | 18,875427 |
| 594 | 7 | 5861 | Steenbras Dam Road | Cape Town | -34,182752 | 18,845777 |
| 594 | 22 | 10994 | N/A | Stellenbosch | -33,96105 | 18,923733 |
| 596 | 1 | 2393 | Harbour Road | Cape Town | -34,053474 | 18,344135 |
| 597 | 1 | 2416 | N/A | Cape Town | -34,077717 | 18,437361 |
| 598 | 3 | 4266 | Emerald Drive | Cape Town | -34,101336 | 18,388967 |
| 598 | 1 | 2421 | Gordon's Bay Road | Cape Town | -34,120728 | 18,836975 |
| 600 | 1 | 2430 | N/A | Cape Town | -33,78533 | 18,761262 |
| 601 | 11 | 7816 | Rustenburg Road | Stellenbosch | -33,911103 | 18,88533 |
| 601 | 1 | 2445 | Seventh Avenue | Cape Town | -34,162825 | 18,399404 |
| 601 | 9 | 6891 | N/A | Cape Town | -34,167408 | 18,94922 |
| 604 | 1 | 2462 | Restio Road | Cape Town | -34,025835 | 18,383561 |
| 604 | 5 | 5189 | Smuts' Track | Cape Town | -33,973797 | 18,420625 |
| 606 | 9 | 6952 | N/A | Cape Town | -34,19343 | 18,832941 |
| 607 | 1 | 2478 | Steenbras Dam Road | Cape Town | -34,15395 | 18,916937 |
| 608 | 1 | 2485 | N/A | Stellenbosch | -33,982647 | 18,811762 |
| 609 | 1 | 2499 | Amandel Street | Cape Town | -33,849705 | 18,667774 |
| 609 | 11 | 7876 | N/A | Stellenbosch | -33,879913 | 18,863108 |
| 611 | 1 | 2507 | Franshoek Street | Cape Town | -33,848374 | 18,641544 |
| 612 | 1 | 2509 | Steenbras Dam Road | Cape Town | -34,167616 | 18,896005 |
| 612 | 5 | 5263 | Volsteedt Street | Cape Town | -34,096061 | 18,828528 |
| 614 | 5 | 5323 | Blaauwklippen Road | Stellenbosch | -33,974505 | 18,860353 |
| 614 | 1 | 2517 | Vlaeberg Road | Stellenbosch | -33,993103 | 18,763568 |
| 614 | 6 | 5755 | N/A | Stellenbosch | -33,892012 | 18,816197 |
| 617 | 1 | 2544 | Regent Road | Cape Town | -33,815918 | 18,503892 |
| 617 | 6 | 5774 | N/A | Cape Town | -33,988779 | 18,390396 |
| 619 | 1 | 2549 | Vryburger Avenue | Cape Town | -33,86057 | 18,539601 |
| 620 | 2 | 3590 | Vlaeberg Road | Stellenbosch | -33,975846 | 18,751989 |
| 621 | 6 | 5848 | Penelope Close | Cape Town | -33,9684 | 18,38197 |
| 622 | 2 | 3637 | Rocket Road | Cape Town | -34,030472 | 18,319283 |
| 622 | 1 | 2584 | N/A | Cape Town | -34,071501 | 18,827679 |
| 624 | 2 | 3649 | Central Road | Cape Town | -34,143054 | 18,426454 |
| 625 | 2 | 3650 | N/A | Cape Town | -34,037693 | 18,320745 |
| 626 | 1 | 2614 | Kuils River Freeway | Cape Town | -33,913774 | 18,668473 |
| 627 | 10 | 7867 | External Red | Cape Town | -34,109428 | 18,952122 |
| 628 | 1 | 2649 | Link Road | Cape Town | -33,813763 | 18,487346 |
| 628 | 3 | 4521 | N/A | Cape Town | -34,115801 | 18,412849 |
| 630 | 1 | 2651 | Vryburger Avenue | Cape Town | -33,858338 | 18,545221 |
| 631 | 2 | 3711 | N/A | Cape Town | -34,001516 | 18,370036 |
| 632 | 8 | 7183 | Faure Marine Drive | Cape Town | -34,193557 | 18,824195 |
| 632 | 6 | 5968 | N/A | Cape Town | -34,172786 | 18,940294 |
| 634 | 7 | 6659 | N/A | Stellenbosch | -33,888305 | 18,828633 |
| 635 | 16 | 11194 | Main Road | Cape Town | -34,25988 | 18,461287 |
| 636 | 4 | 5078 | N/A | Cape Town | -33,960393 | 18,401254 |
| 637 | 16 | 11394 | N/A | Cape Town | -34,267369 | 18,425799 |
| 638 | 4 | 5103 | Waboom Street | Stellenbosch | -33,901947 | 18,841606 |
| 638 | 6 | 6112 | N/A | Stellenbosch | -33,925792 | 18,874472 |
| 640 | 4 | 5126 | N/A | Cape Town | -34,205578 | 18,387199 |
| 640 | 1 | 2712 | N/A | Stellenbosch | -33,976791 | 18,814318 |
| 642 | 1 | 2717 | Highway Road | Cape Town | -34,149299 | 18,435499 |
| 642 | 11 | 8706 | Rustenburg Road | Stellenbosch | -33,910233 | 18,892542 |
| 644 | 6 | 6201 | Bizweni Avenue | Cape Town | -34,088578 | 18,864134 |
| 645 | 7 | 6848 | Three Firs | Cape Town | -33,975182 | 18,38797 |
| 646 | 10 | 8342 | N/A | Stellenbosch | -33,906327 | 18,886726 |
| 647 | 1 | 2742 | Akkerendam Court | Cape Town | -33,55582 | 18,500132 |
| 648 | 9 | 7859 | N/A | Stellenbosch | -33,946125 | 18,898828 |
| 649 | 1 | 2776 | Settlers Way | Cape Town | -33,980109 | 18,580499 |
| 649 | 3 | 4707 | Twelve Apostles | Cape Town | -33,99012 | 18,361471 |
| 651 | 6 | 6441 | N/A | Stellenbosch | -33,970775 | 18,880933 |
| 652 | 1 | 2801 | Watson Way | Stellenbosch | -34,027217 | 18,797267 |
| 653 | 9 | 8037 | Helderzicht Road | Cape Town | -34,072265 | 18,878356 |
| 653 | 12 | 9729 | N/A | Cape Town | -34,058693 | 18,894099 |
| 655 | 3 | 4756 | Devon Vallei | Stellenbosch | -33,903095 | 18,8127 |
| 656 | 1 | 2824 | N/A | Stellenbosch | -33,90254 | 18,772402 |
| 657 | 1 | 2838 | Helderspruit Road | Cape Town | -34,047363 | 18,833466 |
| 658 | 1 | 2842 | Hoerikwaggo Trail | Cape Town | -34,18087 | 18,3938 |
| 658 | 3 | 4805 | Quince Crescent | Cape Town | -34,097666 | 18,380656 |
| 660 | 1 | 2848 | Argyle Street | Cape Town | -33,951103 | 18,380992 |
| 661 | 15 | 11983 | N/A | Stellenbosch | -33,965162 | 18,928767 |
| 662 | 1 | 2860 | Tritonia Avenue | Cape Town | -33,864995 | 18,598145 |
| 663 | 4 | 5458 | N/A | Cape Town | -34,139694 | 18,942042 |
| 664 | 2 | 3937 | N/A | Cape Town | -34,002209 | 18,372818 |
| 665 | 2 | 3962 | Bellevue Road | Cape Town | -34,198295 | 18,45222 |
| 666 | 1 | 2871 | The Meadway Avenue | Cape Town | -33,94465 | 18,3754 |
| 666 | 9 | 8471 | N/A | Cape Town | -34,21867 | 18,887015 |
| 668 | 1 | 2907 | Chestnut Drive | Cape Town | -34,011513 | 18,384919 |
| 669 | 5 | 6164 | N/A | Cape Town | -33,98748 | 18,385969 |
| 670 | 3 | 4887 | N/A | Cape Town | -33,994141 | 18,376554 |
| 671 | 3 | 4889 | Kasteelspoort Path | Cape Town | -33,96237 | 18,390074 |
| 672 | 1 | 2950 | Aurora Street | Cape Town | -33,833445 | 18,623935 |
| 673 | 1 | 2959 | Faure Street | Cape Town | -34,166088 | 18,860125 |
| 673 | 4 | 5683 | N/A | Cape Town | -34,11112 | 18,925993 |
| 675 | 7 | 7767 | Watsonia Trail | Cape Town | -34,042309 | 18,873803 |
| 676 | 3 | 4967 | Jennings Street | Cape Town | -34,096595 | 18,827782 |
| 676 | 4 | 5705 | Victoria Road | Cape Town | -33,982002 | 18,363375 |
| 676 | 1 | 2995 | N/A | Cape Town | -34,151703 | 18,413492 |
| 679 | 1 | 3008 | Clara Anna Fontein | Cape Town | -33,82005 | 18,622615 |
| 680 | 6 | 7076 | Kahler | Stellenbosch | -33,927537 | 18,889567 |
| 680 | 5 | 6448 | Plateau Street | Cape Town | -34,231702 | 18,412142 |
| 682 | 2 | 4181 | Contour Path | Cape Town | -33,95755 | 18,402625 |
| 682 | 1 | 3021 | Main Road | Cape Town | -34,117312 | 18,825937 |
| 684 | 7 | 7855 | Jonkershoek Road | Stellenbosch | -33,941947 | 18,892147 |
| 685 | 4 | 5817 | Koelenhof Road | Stellenbosch | -33,892912 | 18,831558 |
| 686 | 1 | 3081 | Viola Road | Cape Town | -33,813911 | 18,480846 |
| 687 | 2 | 4259 | N/A | Cape Town | -34,127666 | 18,413163 |
| 688 | 5 | 6736 | Blaauwklippen Road | Stellenbosch | -33,98683 | 18,869725 |
| 689 | 1 | 3138 | N/A | Cape Town | -34,07837 | 18,769345 |
| 690 | 8 | 8893 | Jonkershoek Road | Stellenbosch | -33,953062 | 18,906013 |
| 690 | 1 | 3140 | La Motte Street | Cape Town | -34,11232 | 18,849207 |
| 692 | 8 | 9003 | Myrtle Street | Cape Town | -34,072536 | 18,884252 |
| 692 | 5 | 6835 | Reservoir Road | Cape Town | -34,065105 | 18,86834 |
| 692 | 13 | 13140 | N/A | Stellenbosch | -33,973642 | 18,938203 |
| 692 | 1 | 3165 | N/A | Cape Town | -34,192944 | 18,388739 |
| 696 | 3 | 5219 | Main Road | Cape Town | -34,073095 | 18,848563 |
| 696 | 2 | 4328 | Patou Road | Cape Town | -33,862971 | 18,666291 |
| 696 | 7 | 8274 | N/A | Cape Town | -34,087994 | 18,918545 |
| 699 | 6 | 7751 | External Red | Cape Town | -34,104165 | 18,943512 |
| 699 | 1 | 3191 | Ursinia Avenue | Cape Town | -33,854597 | 18,595529 |
| 701 | 5 | 6897 | Markotter | Stellenbosch | -33,935603 | 18,88232 |
| 701 | 3 | 5232 | N/A | Cape Town | -34,212469 | 18,395822 |
| 703 | 1 | 3218 | Parker Street | Cape Town | -33,840149 | 18,628242 |
| 704 | 1 | 3218 | Japhta K. Masemola Road | Cape Town | -34,018189 | 18,637853 |
| 705 | 1 | 3225 | N/A | Cape Town | -34,153839 | 18,389319 |
| 706 | 1 | 3244 | Sandalwood Street | Cape Town | -33,913279 | 18,697992 |
| 707 | 2 | 4464 | N/A | Cape Town | -34,142764 | 18,93463 |
| 708 | 7 | 8688 | N/A | Stellenbosch | -33,878883 | 18,864905 |
| 709 | 1 | 3265 | N/A | Cape Town | -34,16312 | 18,395957 |
| 710 | 1 | 3267 | N/A | Cape Town | -34,084258 | 18,820263 |
| 711 | 1 | 3268 | N/A | Cape Town | -33,815257 | 18,783761 |
| 712 | 2 | 4493 | N/A | Cape Town | -33,8411 | 18,602651 |
| 713 | 1 | 3274 | Coracle Street | Cape Town | -34,129139 | 18,404441 |
| 713 | 3 | 5469 | Pajaro Avenue | Stellenbosch | -33,981324 | 18,851314 |
| 715 | 3 | 5476 | Old Wagon Road | Cape Town | -34,094402 | 18,407007 |
| 716 | 2 | 4539 | N/A | Stellenbosch | -34,015689 | 18,821067 |
| 716 | 5 | 7441 | N/A | Cape Town | -34,237186 | 18,41425 |
| 718 | 10 | 12162 | Jonkershoek Trail | Stellenbosch | -33,968242 | 18,930363 |
| 719 | 1 | 3350 | N/A | Cape Town | -34,165815 | 18,366482 |
| 720 | 2 | 4649 | The Close | Cape Town | -34,133043 | 18,43262 |
| 720 | 13 | 16958 | N/A | Cape Town | -34,029433 | 18,955307 |
| 722 | 2 | 4650 | Malabar Close | Cape Town | -34,098332 | 18,366696 |
| 723 | 1 | 3402 | N/A | Stellenbosch | -33,813126 | 18,816105 |
| 724 | 1 | 3419 | Apple Close | Cape Town | -33,987073 | 18,681646 |
| 725 | 6 | 8482 | External Black | Cape Town | -34,107697 | 18,951913 |
| 726 | 1 | 3426 | Polkadraai Road | Stellenbosch | -33,964148 | 18,744544 |
| 727 | 11 | 14342 | N/A | Stellenbosch | -33,98083 | 18,950188 |
| 727 | 6 | 8540 | N/A | Cape Town | -34,081537 | 18,893997 |
| 729 | 11 | 14629 | N/A | Stellenbosch | -33,98273 | 18,951297 |
| 730 | 1 | 3455 | Kendal Road | Cape Town | -33,859767 | 18,641511 |
| 731 | 1 | 3459 | Eversdal Road | Cape Town | -33,860744 | 18,67539 |
| 732 | 2 | 4732 | Adam Tas Road | Cape Town | -34,061415 | 18,850831 |
| 733 | 1 | 3480 | Kuils River Freeway | Cape Town | -33,904694 | 18,672686 |
| 734 | 4 | 6955 | N/A | Stellenbosch | -33,908663 | 18,873438 |
| 735 | 1 | 3509 | Allen Street | Cape Town | -34,129219 | 18,917886 |
| 736 | 1 | 3518 | Old Main Road | Cape Town | -34,027262 | 18,742833 |
| 737 | 1 | 3529 | Mondeor Road | Cape Town | -34,116636 | 18,889218 |
| 738 | 2 | 4781 | N/A | Cape Town | -34,10339 | 18,417025 |
| 739 | 1 | 3555 | Dinsley Walk | Cape Town | -34,120607 | 18,892772 |
| 740 | 1 | 3561 | N/A | Cape Town | -34,163426 | 18,383806 |
| 741 | 1 | 3563 | Faure Marine Drive | Cape Town | -34,169497 | 18,862378 |
| 741 | 2 | 4805 | Pastorie Street | Cape Town | -34,079012 | 18,843785 |
| 743 | 9 | 12733 | N/A | Cape Town | -34,242987 | 18,859762 |
| 744 | 2 | 4847 | Bill Bezuidenhout Avenue | Cape Town | -33,879488 | 18,632444 |
| 744 | 4 | 7307 | Knorhoek Road | Stellenbosch | -33,871872 | 18,864047 |
| 744 | 7 | 10394 | Olifantsbos Road | Cape Town | -34,257428 | 18,424171 |
| 747 | 5 | 8214 | Millers Point Road | Cape Town | -34,229781 | 18,470684 |
| 747 | 9 | 12789 | Olifantsbos Road | Cape Town | -34,2737 | 18,4555 |
| 749 | 1 | 3608 | Brigantine Avenue | Cape Town | -34,119906 | 18,39708 |
| 749 | 2 | 4861 | Steenbras Dam Road | Cape Town | -34,182086 | 18,846141 |
| 751 | 2 | 4882 | N/A | Cape Town | -34,176044 | 18,925825 |
| 752 | 1 | 3654 | Richmond Street | Cape Town | -34,112919 | 18,866162 |
| 753 | 1 | 3656 | Katzenellenbogen Road | Cape Town | -34,105933 | 18,376633 |
| 754 | 3 | 6300 | Outer Orange Kloof Ring Road | Cape Town | -33,994861 | 18,402265 |
| 755 | 6 | 9802 | N/A | Cape Town | -34,067908 | 18,897033 |
| 756 | 1 | 3715 | Red Hill Road | Cape Town | -34,201709 | 18,390701 |
| 757 | 1 | 3733 | Hoeveld Road | Cape Town | -34,063036 | 18,844939 |
| 757 | 8 | 12975 | N/A | Stellenbosch | -33,965653 | 18,941595 |
| 759 | 2 | 4978 | De Anker Road | Cape Town | -34,146316 | 18,320537 |
| 759 | 6 | 9987 | Delheim Road | Stellenbosch | -33,875362 | 18,886958 |
| 759 | 1 | 3737 | Sir David Baird Drive | Cape Town | -33,787888 | 18,462898 |
| 762 | 1 | 3743 | Altydgedacht Crescent | Cape Town | -33,848572 | 18,625288 |
| 762 | 3 | 6450 | N/A | Cape Town | -34,152778 | 18,95 |
| 762 | 5 | 8737 | N/A | Cape Town | -34,090273 | 18,93207 |
| 765 | 2 | 4990 | Faure Marine Drive | Cape Town | -34,180263 | 18,83053 |
| 766 | 5 | 8859 | Jonkershoek Road | Stellenbosch | -33,948867 | 18,900938 |
| 766 | 6 | 10148 | Olifantsbos Road | Cape Town | -34,254355 | 18,384612 |
| 768 | 2 | 5038 | Egret Street | Cape Town | -34,194206 | 18,375003 |
| 768 | 1 | 3756 | N/A | Stellenbosch | -33,910387 | 18,810595 |
| 770 | 4 | 7834 | N/A | Cape Town | -34,182763 | 18,945245 |
| 770 | 1 | 3758 | N/A | Cape Town | -34,135035 | 18,920873 |
| 772 | 3 | 6599 | N/A | Cape Town | -34,163751 | 18,950188 |
| 773 | 1 | 3766 | Blanc de Noir Street | Cape Town | -33,851174 | 18,602991 |
| 774 | 1 | 3777 | Sir David Baird Drive | Cape Town | -33,797264 | 18,465442 |
| 775 | 1 | 3778 | N/A | Cape Town | -33,566265 | 18,5179 |
| 776 | 4 | 7900 | N/A | Cape Town | -34,206456 | 18,857642 |
| 777 | 1 | 3790 | La Belle Road | Cape Town | -33,892167 | 18,670417 |
| 778 | 3 | 6773 | N/A | Cape Town | -34,046853 | 18,87149 |
| 779 | 3 | 6777 | Granite Close | Cape Town | -34,092098 | 18,878966 |
| 779 | 2 | 5128 | Oriole Road | Cape Town | -34,124899 | 18,43102 |
| 781 | 2 | 5143 | Stellenboschkloof Road | Stellenbosch | -33,942312 | 18,77353 |
| 782 | 6 | 11188 | N/A | Stellenbosch | -33,969203 | 18,921948 |
| 783 | 2 | 5213 | Orion Close | Cape Town | -34,094099 | 18,856256 |
| 783 | 1 | 3860 | Suikerbossie Drive | Cape Town | -34,171996 | 18,850247 |
| 785 | 2 | 5227 | Van Riebeeckshof Road | Cape Town | -33,862261 | 18,629317 |
| 786 | 1 | 3900 | N/A | Stellenbosch | -34,031287 | 18,837215 |
| 787 | 1 | 3900 | N/A | Cape Town | -33,956527 | 18,392889 |
| 788 | 3 | 6962 | N/A | Cape Town | -34,200214 | 18,862164 |
| 789 | 3 | 7036 | N/A | Cape Town | -34,182517 | 18,944192 |
| 790 | 1 | 3973 | N/A | Stellenbosch | -34,008147 | 18,819353 |
| 791 | 3 | 7310 | N/A | Cape Town | -34,181941 | 18,946082 |
| 792 | 1 | 4085 | Ixia Road | Cape Town | -33,869064 | 18,498068 |
| 793 | 4 | 8855 | Jonkershoek Road | Stellenbosch | -33,941105 | 18,905403 |
| 794 | 2 | 5549 | Stork Street | Cape Town | -33,544383 | 18,519265 |
| 795 | 2 | 5558 | N/A | Stellenbosch | -33,999655 | 18,838709 |
| 796 | 4 | 8887 | N/A | Stellenbosch | -33,929927 | 18,904786 |
| 797 | 1 | 4134 | Helderberg Farm 4x4 Trail | Stellenbosch | -34,040776 | 18,841004 |
| 798 | 1 | 4142 | Main Road | Cape Town | -34,103746 | 18,84457 |
| 799 | 1 | 4153 | N/A | Stellenbosch | -33,956812 | 18,741858 |
| 800 | 3 | 7577 | N/A | Cape Town | -34,164841 | 18,959174 |
| 800 | 2 | 5644 | N/A | Cape Town | -34,132162 | 18,940301 |
| 802 | 1 | 4180 | Langverwacht Road | Cape Town | -33,930098 | 18,726556 |
| 803 | 1 | 4209 | Alleyne Yeld Crescent | Cape Town | -34,131188 | 18,412291 |
| 804 | 4 | 9284 | N/A | Cape Town | -34,088512 | 18,937726 |
| 805 | 4 | 9393 | Olifantsbos Road | Cape Town | -34,256133 | 18,422269 |
| 806 | 3 | 7803 | Botterberg Road | Cape Town | -33,707698 | 18,547617 |
| 806 | 1 | 4256 | N/A | Cape Town | -33,881052 | 18,659944 |
| 808 | 1 | 4262 | Marine Drive | Cape Town | -33,831186 | 18,481771 |
| 808 | 5 | 11727 | N/A | Cape Town | -34,055311 | 18,910286 |
| 810 | 2 | 5778 | Nthombeni Way | Cape Town | -34,088889 | 18,3825 |
| 811 | 1 | 4264 | High Riding Drive | Cape Town | -34,122786 | 18,925367 |
| 811 | 2 | 5793 | Hillcrest Estate | Cape Town | -33,830142 | 18,590851 |
| 813 | 1 | 4286 | Mahanyele Street | Cape Town | -34,11633 | 18,914412 |
| 814 | 1 | 4320 | Jip De Jager Drive | Cape Town | -33,873572 | 18,618515 |
| 815 | 1 | 4327 | Kendal Road | Cape Town | -33,864137 | 18,654767 |
| 816 | 2 | 5859 | Ottawa Avenue | Cape Town | -33,971282 | 18,374654 |
| 817 | 4 | 9903 | N/A | Cape Town | -34,088415 | 18,952097 |
| 818 | 1 | 4350 | N/A | Cape Town | -34,174405 | 18,363671 |
| 819 | 3 | 8150 | N/A | Stellenbosch | -33,995438 | 18,879078 |
| 820 | 6 | 15580 | N/A | Stellenbosch | -33,9831 | 18,954033 |
| 821 | 1 | 4460 | N/A | Cape Town | -34,157524 | 18,328522 |
| 822 | 3 | 8324 | Davy Street | Stellenbosch | -33,918827 | 18,892149 |
| 823 | 1 | 4484 | N/A | Stellenbosch | -33,987417 | 18,829708 |
| 824 | 1 | 4492 | N/A | Cape Town | -34,187462 | 18,892913 |
| 825 | 1 | 4525 | N/A | Stellenbosch | -33,81665 | 18,82949 |
| 826 | 3 | 8568 | Kerk Street | Cape Town | -33,506774 | 18,471626 |
| 826 | 2 | 6342 | Stellenrust Road | Stellenbosch | -33,988705 | 18,854838 |
| 826 | 5 | 13685 | N/A | Stellenbosch | -33,984897 | 18,944088 |
| 829 | 1 | 4562 | Boschendal Street | Cape Town | -33,854303 | 18,610313 |
| 829 | 5 | 13760 | Cape of Good Hope Drive | Cape Town | -34,279503 | 18,454058 |
| 829 | 2 | 6433 | N/A | Cape Town | -34,097814 | 18,88484 |
| 832 | 1 | 4637 | N/A | Cape Town | -34,02865 | 18,315864 |
| 833 | 1 | 4654 | Helderberg Farm 4x4 Trail | Stellenbosch | -34,03142 | 18,838829 |
| 833 | 5 | 14568 | N/A | Cape Town | -34,055523 | 18,956884 |
| 835 | 1 | 4657 | Lourens River Road | Cape Town | -34,105981 | 18,82463 |
| 836 | 1 | 4666 | Mervyn Road | Cape Town | -34,131246 | 18,415956 |
| 837 | 2 | 6727 | N/A | Cape Town | -34,052489 | 18,873008 |
| 838 | 1 | 4693 | Twelve Apostles | Cape Town | -33,989888 | 18,360017 |
| 839 | 1 | 4706 | Graaff Avenue | Cape Town | -33,882815 | 18,488907 |
| 840 | 4 | 12029 | N/A | Cape Town | -34,060958 | 18,919983 |
| 841 | 1 | 4716 | Sesithathu Street | Stellenbosch | -33,911187 | 18,850055 |
| 842 | 3 | 9274 | N/A | Stellenbosch | -33,919693 | 18,902383 |
| 843 | 1 | 4736 | Victoria Road | Cape Town | -33,992118 | 18,350523 |
| 844 | 1 | 4739 | Tygerberg Valley Road | Cape Town | -33,86484 | 18,631255 |
| 844 | 2 | 6831 | N/A | Cape Town | -33,976207 | 18,374449 |
| 846 | 3 | 9564 | Upper Blaauwklippen Road | Stellenbosch | -33,9996 | 18,884725 |
| 846 | 4 | 12569 | N/A | Cape Town | -34,245887 | 18,870883 |
| 848 | 1 | 4826 | Dassenberg Street | Cape Town | -33,543535 | 18,507563 |
| 849 | 1 | 4844 | Waterfront Road | Cape Town | -33,874052 | 18,626726 |
| 850 | 2 | 7057 | N/A | Cape Town | -33,981587 | 18,404762 |
| 851 | 3 | 10010 | Helshoogte Road | Stellenbosch | -33,922397 | 18,910022 |
| 852 | 2 | 7325 | N/A | Cape Town | -34,203203 | 18,923953 |
| 852 | 4 | 14084 | N/A | Cape Town | -34,052023 | 18,953496 |
| 854 | 1 | 4894 | Simonstown to Smitswinkel via Swartkop | Cape Town | -34,212073 | 18,442185 |
| 855 | 2 | 7393 | Plateau Street | Cape Town | -34,23856 | 18,42134 |
| 856 | 2 | 7463 | N/A | Stellenbosch | -33,989671 | 18,870876 |
| 857 | 1 | 4923 | Via Lucia Street | Cape Town | -34,118458 | 18,92101 |
| 858 | 3 | 10882 | N/A | Stellenbosch | -33,935923 | 18,923998 |
| 859 | 1 | 4979 | N/A | Stellenbosch | -33,878055 | 18,812442 |
| 860 | 4 | 15383 | Sosyskloof-Swartboskloof Contour | Stellenbosch | -33,999724 | 18,948712 |
| 861 | 3 | 11376 | N/A | Cape Town | -34,02295 | 18,902158 |
| 862 | 2 | 7758 | N/A | Stellenbosch | -33,86932 | 18,872647 |
| 862 | 1 | 5059 | N/A | Cape Town | -34,164438 | 18,332474 |
| 864 | 4 | 15972 | N/A | Cape Town | -34,028798 | 18,951425 |
| 865 | 4 | 16290 | Sosyskloof-Swartboskloof Contour | Stellenbosch | -34,000214 | 18,956537 |
| 865 | 2 | 7781 | N/A | Stellenbosch | -33,979002 | 18,890694 |
| 867 | 1 | 5107 | N/A | Cape Town | -33,839706 | 18,606516 |
| 868 | 1 | 5142 | Aerodrome Road | Cape Town | -34,097233 | 18,844786 |
| 869 | 1 | 5191 | Joubert Street | Cape Town | -34,104652 | 18,817266 |
| 870 | 2 | 7912 | N/A | Cape Town | -34,088043 | 18,917185 |
| 871 | 2 | 7931 | Woodie's Walk | Cape Town | -34,038471 | 18,873487 |
| 872 | 4 | 20362 | Kurktrekker | Stellenbosch | -34,006146 | 18,995531 |
| 872 | 3 | 12128 | N/A | Cape Town | -34,266 | 18,463 |
| 874 | 2 | 8049 | N/A | Stellenbosch | -33,92895 | 18,898175 |
| 875 | 2 | 8121 | Vergelegen Avenue | Cape Town | -34,082238 | 18,887613 |
| 876 | 1 | 5350 | N/A | Cape Town | -34,179639 | 18,367044 |
| 877 | 3 | 12484 | N/A | Cape Town | -34,246887 | 18,873373 |
| 878 | 2 | 8224 | Malanshoogte Road | Cape Town | -33,749344 | 18,617254 |
| 878 | 1 | 5367 | Rocklands Road | Cape Town | -34,211464 | 18,460874 |
| 880 | 1 | 5369 | Clovelly Road | Cape Town | -34,118701 | 18,418589 |
| 881 | 1 | 5401 | Camel Rock Road | Cape Town | -34,202672 | 18,377822 |
| 882 | 1 | 5410 | Simonstown to Smitswinkel via Swartkop | Cape Town | -34,215849 | 18,45767 |
| 882 | 3 | 12751 | N/A | Cape Town | -34,046145 | 18,92229 |
| 884 | 1 | 5441 | Yellowwood Drive | Cape Town | -34,085433 | 18,387578 |
| 885 | 1 | 5453 | N/A | Cape Town | -34,159251 | 18,940083 |
| 886 | 1 | 5471 | N/A | Cape Town | -34,19371 | 18,883153 |
| 887 | 3 | 13378 | N/A | Stellenbosch | -33,978058 | 18,944847 |
| 888 | 3 | 13588 | Old Malmesbury Road | Cape Town | -33,677226 | 18,605813 |
| 888 | 2 | 8560 | N/A | Cape Town | -34,162051 | 18,964507 |
| 890 | 3 | 13717 | N/A | Cape Town | -34,055801 | 18,956434 |
| 891 | 3 | 13967 | N/A | Stellenbosch | -33,963292 | 18,94523 |
| 891 | 1 | 5588 | N/A | Cape Town | -33,818236 | 18,578847 |
| 893 | 3 | 14120 | N/A | Stellenbosch | -33,973587 | 18,950928 |
| 893 | 1 | 5630 | N/A | Cape Town | -34,176872 | 18,927398 |
| 895 | 2 | 8811 | Lourensford Road | Cape Town | -34,067802 | 18,889989 |
| 896 | 1 | 5691 | N/A | Cape Town | -34,194224 | 18,872031 |
| 897 | 1 | 5699 | N/A | Cape Town | -34,226653 | 18,405915 |
| 898 | 2 | 8908 | N/A | Cape Town | -34,034722 | 18,888647 |
| 899 | 1 | 5707 | Charel Uys Drive | Cape Town | -33,52746 | 18,49434 |
| 899 | 3 | 15102 | N/A | Cape Town | -34,054176 | 18,966074 |
| 901 | 1 | 5720 | Steenbras Dam Road | Cape Town | -34,187352 | 18,850899 |
| 902 | 1 | 5727 | Silverboom Kloof Road | Cape Town | -34,055059 | 18,860025 |
| 902 | 2 | 9027 | N/A | Stellenbosch | -33,924982 | 18,904635 |
| 904 | 2 | 9182 | Rustenburg Road | Stellenbosch | -33,905317 | 18,894713 |
| 905 | 1 | 5816 | Military Road | Cape Town | -33,752155 | 18,467287 |
| 905 | 3 | 15908 | N/A | Stellenbosch | -33,991389 | 18,954167 |
| 907 | 2 | 9485 | Vergelegen Avenue | Cape Town | -34,076447 | 18,891388 |
| 908 | 2 | 9488 | N/A | Cape Town | -34,22059 | 18,880942 |
| 909 | 2 | 9600 | N/A | Cape Town | -34,225278 | 18,865 |
| 910 | 3 | 17791 | Witbrug | Stellenbosch | -33,993907 | 18,975251 |
| 911 | 1 | 5940 | Kromme Rhee Road | Stellenbosch | -33,869323 | 18,848573 |
| 911 | 2 | 9813 | N/A | Cape Town | -34,21974 | 18,85978 |
| 913 | 2 | 9851 | Main Road | Cape Town | -34,24411 | 18,473949 |
| 914 | 3 | 18742 | Berg River Neck Trail | Stellenbosch | -33,99577 | 18,98099 |
| 914 | 1 | 5999 | Helderberg Farm 4x4 Trail | Cape Town | -34,037844 | 18,861117 |
| 916 | 1 | 6096 | N/A | Cape Town | -34,225651 | 18,394943 |
| 917 | 2 | 10119 | N/A | Cape Town | -34,026073 | 18,898256 |
| 918 | 1 | 6139 | Sea View Road | Cape Town | -34,071837 | 18,861205 |
| 919 | 1 | 6280 | Delvera Mountain Bike Trail | Stellenbosch | -33,852086 | 18,857672 |
| 920 | 2 | 10665 | N/A | Cape Town | -34,098416 | 18,96483 |
| 921 | 1 | 6317 | N/A | Cape Town | -34,080076 | 18,366541 |
| 922 | 2 | 10774 | N/A | Cape Town | -34,259288 | 18,389165 |
| 923 | 1 | 6433 | N/A | Cape Town | -34,14708 | 18,947361 |
| 924 | 2 | 11080 | N/A | Cape Town | -34,257773 | 18,386709 |
| 924 | 1 | 6455 | N/A | Cape Town | -34,140732 | 18,952412 |
| 926 | 1 | 6543 | N/A | Cape Town | -34,098664 | 18,893566 |
| 927 | 1 | 6566 | Malan Street | Cape Town | -34,078793 | 18,856826 |
| 928 | 2 | 11424 | N/A | Cape Town | -34,269638 | 18,414717 |
| 928 | 1 | 6658 | N/A | Cape Town | -34,202701 | 18,867309 |
| 930 | 2 | 11452 | Olifantsbos Road | Cape Town | -34,264288 | 18,434703 |
| 931 | 2 | 11472 | Old Boland Trail | Cape Town | -34,094294 | 18,977914 |
| 932 | 2 | 11602 | Main Road | Cape Town | -34,259009 | 18,471031 |
| 933 | 2 | 11729 | N/A | Cape Town | -34,062011 | 18,916199 |
| 934 | 2 | 11753 | N/A | Cape Town | -34,048175 | 18,91107 |
| 935 | 1 | 6795 | N/A | Stellenbosch | -33,906362 | 18,871755 |
| 935 | 2 | 11755 | N/A | Cape Town | -34,23751 | 18,862844 |
| 937 | 1 | 6810 | Main Road | Cape Town | -34,225167 | 18,467253 |
| 937 | 2 | 11822 | N/A | Cape Town | -34,269697 | 18,455025 |
| 939 | 2 | 11859 | N/A | Stellenbosch | -33,874754 | 18,904334 |
| 940 | 2 | 12013 | N/A | Cape Town | -34,234794 | 18,851495 |
| 941 | 2 | 12023 | N/A | Cape Town | -34,267527 | 18,387397 |
| 942 | 1 | 6900 | N/A | Stellenbosch | -33,954093 | 18,882962 |
| 943 | 1 | 6974 | Dassenberg Drive | Cape Town | -33,627438 | 18,439869 |
| 944 | 2 | 12727 | N/A | Cape Town | -34,055556 | 18,926944 |
| 945 | 1 | 7177 | West Coast Road | Cape Town | -33,631017 | 18,447244 |
| 946 | 1 | 7257 | N/A | Cape Town | -34,091163 | 18,890616 |
| 946 | 2 | 12852 | N/A | Cape Town | -34,035325 | 18,92222 |
| 948 | 2 | 13290 | N/A | Cape Town | -34,054722 | 18,927778 |
| 949 | 1 | 7491 | N/A | Stellenbosch | -33,981605 | 18,877267 |
| 950 | 1 | 7529 | N/A | Stellenbosch | -33,899063 | 18,870154 |
| 951 | 1 | 7542 | N/A | Stellenbosch | -33,901856 | 18,877183 |
| 952 | 1 | 7547 | Mamre Road | Cape Town | -33,719009 | 18,510457 |
| 953 | 1 | 7619 | Olifantsbos Road | Cape Town | -34,236353 | 18,397902 |
| 954 | 2 | 14554 | Circular Drive | Cape Town | -34,292294 | 18,444465 |
| 954 | 1 | 7710 | N/A | Cape Town | -34,189871 | 18,936526 |
| 956 | 2 | 15436 | N/A | Cape Town | -34,04343 | 18,96115 |
| 957 | 1 | 7830 | N/A | Cape Town | -33,560637 | 18,556303 |
| 958 | 2 | 16348 | N/A | Cape Town | -34,011085 | 18,947136 |
| 959 | 1 | 7920 | N/A | Cape Town | -34,235702 | 18,396708 |
| 960 | 2 | 17150 | N/A | Cape Town | -34,025052 | 18,958294 |
| 961 | 1 | 8041 | N/A | Stellenbosch | -33,964055 | 18,893146 |
| 962 | 2 | 18393 | Circular Drive | Cape Town | -34,322602 | 18,421085 |
| 963 | 2 | 18662 | Platboom Road | Cape Town | -34,317494 | 18,453013 |
| 964 | 1 | 8214 | Helderberg Dome | Cape Town | -34,033034 | 18,875463 |
| 965 | 1 | 8236 | N/A | Cape Town | -33,511801 | 18,51174 |
| 966 | 1 | 8246 | N/A | Stellenbosch | -33,996862 | 18,871447 |
| 967 | 1 | 8435 | Olifantsbos Road | Cape Town | -34,24735 | 18,41186 |
| 968 | 1 | 8446 | N/A | Cape Town | -34,157162 | 18,970838 |
| 969 | 1 | 8494 | N/A | Cape Town | -34,095392 | 18,944261 |
| 970 | 1 | 8676 | N/A | Stellenbosch | -33,976111 | 18,891944 |
| 971 | 1 | 8704 | External Black | Cape Town | -34,114649 | 18,955092 |
| 972 | 1 | 8729 | Triplets Way | Cape Town | -34,059608 | 18,890255 |
| 973 | 1 | 8873 | Upper Blaauwklippen Road | Stellenbosch | -33,996525 | 18,874783 |
| 974 | 1 | 8950 | N/A | Stellenbosch | -33,893892 | 18,889783 |
| 975 | 1 | 9071 | Muratie Road | Stellenbosch | -33,870471 | 18,875923 |
| 976 | 1 | 9145 | Koeberg 4x4 | Cape Town | -33,639845 | 18,425199 |
| 977 | 1 | 9245 | Melkbosstrand Road | Cape Town | -33,725952 | 18,461038 |
| 978 | 1 | 9404 | N/A | Stellenbosch | -33,85611 | 18,887777 |
| 979 | 1 | 9471 | N/A | Cape Town | -34,221111 | 18,882222 |
| 980 | 1 | 9568 | Main Road | Cape Town | -33,502194 | 18,473244 |
| 981 | 1 | 9612 | N/A | Cape Town | -34,085398 | 18,930818 |
| 982 | 1 | 9648 | Delheim Road | Stellenbosch | -33,869378 | 18,887812 |
| 983 | 1 | 10006 | Jonkershoek Road | Stellenbosch | -33,960147 | 18,91573 |
| 984 | 1 | 10218 | Beach Road | Cape Town | -33,722284 | 18,447324 |
| 985 | 1 | 10285 | Upper Blaauwklippen Road | Stellenbosch | -34,004739 | 18,888363 |
| 986 | 1 | 10393 | Olifantsbos Road | Cape Town | -34,263129 | 18,418962 |
| 987 | 1 | 10626 | N/A | Cape Town | -34,068646 | 18,90084 |
| 988 | 1 | 10716 | Main Road | Cape Town | -34,24949 | 18,474988 |
| 989 | 1 | 10727 | N/A | Cape Town | -34,054344 | 18,906954 |
| 990 | 1 | 10862 | Plateau Street | Cape Town | -34,258806 | 18,459368 |
| 991 | 1 | 10917 | N/A | Stellenbosch | -33,874204 | 18,89864 |
| 992 | 1 | 10942 | N/A | Stellenbosch | -33,905583 | 18,915222 |
| 993 | 1 | 11184 | N/A | Cape Town | -33,665133 | 18,440656 |
| 994 | 1 | 11412 | N/A | Stellenbosch | -33,898554 | 18,909332 |
| 995 | 1 | 11746 | N/A | Cape Town | -33,669379 | 18,44374 |
| 996 | 1 | 12054 | N/A | Cape Town | -34,032022 | 18,910202 |
| 997 | 1 | 12281 | West Coast Road | Cape Town | -33,540646 | 18,364704 |
| 998 | 1 | 12352 | N/A | Cape Town | -34,056194 | 18,928898 |
| 999 | 1 | 12419 | N/A | Stellenbosch | -33,97767 | 18,935953 |
| 1000 | 1 | 12447 | N/A | Cape Town | -34,27528 | 18,434953 |
| 1001 | 1 | 12810 | N/A | Stellenbosch | -33,874896 | 18,915155 |
| 1002 | 1 | 13004 | N/A | Cape Town | -33,667128 | 18,766659 |
| 1003 | 1 | 13714 | N/A | Cape Town | -34,25028 | 18,85538 |
| 1004 | 1 | 14240 | N/A | Cape Town | -34,023726 | 18,92976 |
| 1005 | 1 | 14284 | N/A | Cape Town | -34,05949 | 18,9639 |
| 1006 | 1 | 14980 | N/A | Cape Town | -34,046737 | 18,950644 |
| 1007 | 1 | 15104 | N/A | Stellenbosch | -33,967917 | 18,954376 |
| 1008 | 1 | 15206 | N/A | Cape Town | -34,019194 | 18,9375 |
| 1009 | 1 | 15416 | N/A | Cape Town | -34,294895 | 18,410745 |
| 1010 | 1 | 15436 | N/A | Cape Town | -34,297286 | 18,434798 |
| 1011 | 1 | 15942 | N/A | Cape Town | -34,04161 | 18,964252 |
| 1012 | 1 | 16320 | N/A | Cape Town | -34,034802 | 18,95793 |
| 1013 | 1 | 16694 | N/A | Cape Town | -34,301857 | 18,459133 |
| 1014 | 1 | 16878 | Old Malmesbury Road | Cape Town | -33,643312 | 18,624691 |
| 1015 | 1 | 17393 | Circular Drive | Cape Town | -34,311466 | 18,417732 |
| 1016 | 1 | 18519 | Platboom Road | Cape Town | -34,321897 | 18,446635 |
| 1017 | 1 | 19068 | Second Waterfall path | Stellenbosch | -33,99985 | 18,989475 |
| 1018 | 1 | 20008 | Second Waterfall path | Stellenbosch | -34,002283 | 18,993849 |
| 1019 | 1 | 21904 | Berg River Neck Trail | Stellenbosch | -33,999104 | 19,01112 |
| 1020 | 1 | 22979 | Cape Point Road | Cape Town | -34,351541 | 18,470539 |
| 1021 | 1 | 23955 | Cape of Good Hope | Cape Town | -34,356758 | 18,474741 |

## **Table S3.** Roads per priority km^2^ grid cell for polyphagous shot hole borer (*Euwallacea* *fornicatus*) visual surveys based on *Acer negundo* densities (per km^2^) and their proximity to plant biomass sites (PBS). “N/A” = no roads within the km^2^ grid cell.

| **Priority km^2^ grid cell** | **Reproductive host/km^2^** | **Distance to nearest PBS (m)** | **Road name** | **Jurisdiction** | **Latitude** | **Longitude** |
| --- | --- | --- | --- | --- | --- | --- |
| 1 | 12 | 29 | Main Road | Cape Town | -34,001203 | 18,47322 |
| 2 | 7 | 8 | Strand Street | Cape Town | -33,925839 | 18,418354 |
| 3 | 49 | 1029 | Peffers Street | Cape Town | -33,995837 | 18,472097 |
| 4 | 10 | 967 | Waterloo Road | Cape Town | -34,008758 | 18,463812 |
| 5 | 22 | 1412 | Paradise View Road | Cape Town | -33,989031 | 18,466244 |
| 6 | 8 | 1053 | N/A | Cape Town | -33,958059 | 18,47251 |
| 7 | 5 | 968 | Waterloo Road | Cape Town | -33,998024 | 18,466829 |
| 8 | 2 | 53 | Klipfontein Road | Cape Town | -33,953143 | 18,479952 |
| 9 | 20 | 2029 | Chichester Road | Cape Town | -33,988314 | 18,476349 |
| 10 | 6 | 1394 | Church Street | Cape Town | -33,966137 | 18,472903 |
| 11 | 2 | 855 | Simon van der Stel Freeway | Cape Town | -34,018956 | 18,456277 |
| 12 | 2 | 855 | Tortelduif Street | Stellenbosch | -33,940455 | 18,83097 |
| 13 | 2 | 914 | N/A | Cape Town | -33,962422 | 18,492934 |
| 13 | 3 | 1056 | Settlers Way | Cape Town | -33,949426 | 18,473178 |
| 15 | 2 | 916 | N/A | Cape Town | -33,986977 | 18,464779 |
| 15 | 16 | 2247 | Stanhope Road | Cape Town | -33,96599 | 18,48162 |
| 17 | 1 | 101 | Settlers Way | Cape Town | -33,949012 | 18,478153 |
| 18 | 7 | 2029 | Belvedere Road | Cape Town | -34,026274 | 18,43582 |
| 18 | 1 | 351 | Sohland Avenue | Cape Town | -33,987517 | 18,480673 |
| 20 | 1 | 722 | Cardiff Street | Cape Town | -33,813789 | 18,69464 |
| 21 | 1 | 798 | Woodgate Road | Cape Town | -34,018208 | 18,472045 |
| 22 | 27 | 3029 | Campground Road | Cape Town | -33,97668 | 18,474495 |
| 23 | 3 | 1914 | Kromboom Road | Cape Town | -33,969124 | 18,492201 |
| 23 | 1 | 874 | Uitsig Crescent | Cape Town | -33,837667 | 18,668258 |
| 25 | 3 | 1968 | Edinburgh Drive | Cape Town | -34,000134 | 18,451861 |
| 26 | 16 | 3179 | Campground Road | Cape Town | -33,977758 | 18,467578 |
| 27 | 4 | 2185 | Campground Road | Cape Town | -33,974148 | 18,456434 |
| 27 | 27 | 3611 | Paradise Road | Cape Town | -33,967807 | 18,469943 |
| 29 | 5 | 2826 | N/A | Cape Town | -33,985895 | 18,459153 |
| 29 | 1 | 1215 | Government Avenue | Cape Town | -33,930042 | 18,412673 |
| 31 | 4 | 2807 | N/A | Cape Town | -33,995421 | 18,449971 |
| 31 | 8 | 3141 | Klaassens Road | Cape Town | -33,967917 | 18,463464 |
| 31 | 1 | 1368 | Settlers Way | Cape Town | -33,938777 | 18,46908 |
| 34 | 1 | 1371 | Park Road | Cape Town | -34,011322 | 18,466713 |
| 35 | 9 | 3595 | Rose Street | Cape Town | -33,986764 | 18,442612 |
| 35 | 4 | 2866 | Saffraan | Stellenbosch | -33,95022 | 18,849378 |
| 37 | 2 | 2089 | Baden Powell Drive | Stellenbosch | -34,016793 | 18,445539 |
| 37 | 1 | 1557 | Constantia Main Road | Cape Town | -33,973133 | 18,784495 |
| 39 | 1 | 1855 | Flamingo | Stellenbosch | -33,95415 | 18,853978 |
| 39 | 4 | 2942 | Lovell | Stellenbosch | -33,940953 | 18,842503 |
| 41 | 10 | 4456 | Rose Street | Cape Town | -33,982626 | 18,439847 |
| 41 | 1 | 1915 | Sandown Road | Cape Town | -33,971663 | 18,486792 |
| 43 | 3 | 2915 | Stanford Road | Cape Town | -33,976653 | 18,482229 |
| 43 | 1 | 1932 | Vissershokpad | Cape Town | -33,831454 | 18,638418 |
| 45 | 1 | 2061 | Southern Cross Drive | Cape Town | -34,012055 | 18,432441 |
| 46 | 1 | 2092 | Frans Conradie Drive | Cape Town | -33,894987 | 18,61832 |
| 47 | 6 | 4732 | Adam Tas Road | Cape Town | -34,062609 | 18,853332 |
| 47 | 1 | 2220 | Edinburgh Drive | Cape Town | -33,991077 | 18,458811 |
| 49 | 1 | 2544 | Regent Road | Cape Town | -33,815918 | 18,503892 |
| 50 | 3 | 4240 | Union Avenue | Cape Town | -33,979737 | 18,44736 |
| 51 | 1 | 2855 | Culemborg | Stellenbosch | -33,941813 | 18,85147 |
| 52 | 2 | 3603 | Union Avenue | Cape Town | -33,973503 | 18,456737 |
| 53 | 1 | 2953 | Merriman Avenue | Stellenbosch | -33,929622 | 18,852739 |
| 54 | 3 | 4914 | Hospitaal | Stellenbosch | -33,93577 | 18,865675 |
| 55 | 1 | 3312 | Strand Road | Stellenbosch | -33,970937 | 18,4497 |
| 55 | 2 | 4482 | Union Avenue | Cape Town | -33,960883 | 18,854308 |
| 57 | 1 | 3351 | Adam Tas Street | Stellenbosch | -33,928952 | 18,852788 |
| 58 | 1 | 3727 | Waveren Avenue | Cape Town | -34,055176 | 18,84609 |
| 59 | 2 | 5731 | Oldenland Road | Cape Town | -34,060722 | 18,863177 |
| 60 | 1 | 4334 | Main Road | Cape Town | -34,071181 | 18,841411 |
| 61 | 1 | 4756 | Devon Vallei | Stellenbosch | -33,903603 | 18,816045 |
| 62 | 1 | 4880 | Impala Road | Cape Town | -34,065391 | 18,853414 |
| 63 | 1 | 5219 | Main Road | Cape Town | -34,071036 | 18,854451 |
| 64 | 1 | 5854 | Reservoir Road | Cape Town | -34,064721 | 18,86307 |
| 65 | 1 | 5904 | Jannasch | Stellenbosch | -33,93502 | 18,878758 |

## **Table S4**. A list of plant biomass sites across the study area for site-specific monitoring for polyphagous shot hole borer (*Euwallacea* *fornicatus*) infestations.

| **Facility type** | **Jurisdiction** | **Latitude** | **Longitude** |
| --- | --- | --- | --- |
| Arborist facilities | Cape Town | 18,5267300 | -34,0092900 |
|  | Cape Town | 18,5257100 | -34,0041200 |
|  | Cape Town | 18,7377100 | -33,8930700 |
|  | Cape Town | 18,4308600 | -33,9923100 |
|  | Cape Town | 18,3659500 | -34,0176900 |
|  | Cape Town | 18,4345600 | -34,0614300 |
| Firewood distributors | Cape Town | 18,8385500 | -34,0724300 |
|  | Cape Town | 18,5935400 | -33,9188000 |
|  | Cape Town | 18,5283100 | -33,9515900 |
|  | Cape Town | 18,5154000 | -33,9640700 |
|  | Cape Town | 18,5980400 | -33,9032900 |
|  | Cape Town | 18,6351900 | -33,8964600 |
|  | Cape Town | 18,6781000 | -33,8841500 |
|  | Cape Town | 18,7129100 | -33,8909700 |
|  | Cape Town | 18,6277300 | -33,8919400 |
|  | Cape Town | 18,5202900 | -33,9559200 |
|  | Cape Town | 18,6886700 | -33,9564700 |
|  | Cape Town | 18,6885700 | -33,8568600 |
|  | Stellenbosch | 18,5490100 | -33,9122700 |
|  | Cape Town | 18,8115100 | -34,0653600 |
|  | Cape Town | 18,8401100 | -34,0943800 |
|  | Stellenbosch | 18,8525700 | -34,0960700 |
|  | Cape Town | 18,8396500 | -34,1198800 |
|  | Cape Town | 18,8529700 | -34,1428900 |
|  | Cape Town | 18,8531000 | -34,1047300 |
|  | Stellenbosch | 18,7345200 | -34,0251700 |
|  | Stellenbosch | 18,8353200 | -33,9332100 |
|  | Stellenbosch | 18,6892000 | -33,9736100 |
|  | Stellenbosch | 18,6879100 | -33,9323900 |
|  | Cape Town | 18,4892300 | -33,9363600 |
|  | Cape Town | 18,4682800 | -33,9648200 |
|  | Cape Town | 18,4692300 | -33,9943500 |
|  | Cape Town | 18,4690300 | -34,0245900 |
|  | Cape Town | 18,4767400 | -34,0400200 |
|  | Cape Town | 18,4579800 | -34,0696100 |
|  | Stellenbosch | 18,4557900 | -34,0807500 |
|  | Cape Town | 18,4892300 | -33,9363600 |
|  | Cape Town | 18,4892300 | -33,9363600 |
|  | Cape Town | 18,4569700 | -33,7252400 |
| General waste facility | Stellenbosch | 18,8204400 | -33,9465600 |
|  | Cape Town | 18,5624000 | -33,9568600 |
|  | Cape Town | 18,7373500 | -33,8382300 |
|  | Cape Town | 18,5610300 | -33,9166600 |
|  | Cape Town | 18,6569500 | -33,8296900 |
|  | Cape Town | 18,6054800 | -33,9285400 |
|  | Cape Town | 18,5346900 | -34,0368500 |
|  | Cape Town | 18,6352100 | -33,9514500 |
|  | Cape Town | 18,6421400 | -33,9781300 |
|  | Cape Town | 18,5479400 | -33,8933800 |
|  | Cape Town | 18,7481300 | -34,0595500 |
|  | Cape Town | 18,6965100 | -34,0156600 |
|  | Cape Town | 18,5149300 | -33,9156700 |
|  | Cape Town | 18,5809000 | -33,9110000 |
|  | Cape Town | 18,5268200 | -33,8292900 |
|  | Cape Town | 18,8872400 | -34,1489000 |
|  | Cape Town | 18,6479000 | -34,0517400 |
|  | Cape Town | 18,5162500 | -33,9894600 |
|  | Cape Town | 18,5962600 | -34,0664700 |
|  | Cape Town | 18,6484300 | -33,9307000 |
|  | Cape Town | 18,7390900 | -33,8387300 |
|  | Cape Town | 18,6490100 | -33,9320000 |
|  | Cape Town | 18,5956000 | -33,9058400 |
|  | Cape Town | 18,5248200 | -33,8364100 |
|  | Cape Town | 18,7047700 | -33,8773400 |
|  | Cape Town | 18,7152300 | -33,9831300 |
|  | Cape Town | 18,8003600 | -34,0594300 |
|  | Cape Town | 18,5921800 | -34,0595200 |
|  | Cape Town | 18,5914100 | -34,0594700 |
|  | Cape Town | 18,5724700 | -34,0054300 |
|  | Cape Town | 18,6094300 | -33,9947200 |
|  | Cape Town | 18,5716700 | -33,9094600 |
|  | Cape Town | 18,6420600 | -33,9791200 |
|  | Cape Town | 18,5339100 | -34,0365000 |
|  | Cape Town | 18,6805300 | -34,0472900 |
|  | Cape Town | 18,6194500 | -34,0490500 |
|  | Cape Town | 18,6530700 | -33,9301300 |
|  | Cape Town | 18,7272200 | -33,8556900 |
|  | Cape Town | 18,6828900 | -33,9367000 |
|  | Cape Town | 18,6592400 | -33,8294300 |
|  | Cape Town | 18,7186900 | -33,8610000 |
|  | Cape Town | 18,5290200 | -33,9997700 |
|  | Cape Town | 18,6081800 | -33,9956400 |
|  | Cape Town | 18,6605900 | -33,8278800 |
|  | Cape Town | 18,6057300 | -33,9943700 |
|  | Cape Town | 18,4451200 | -34,0300700 |
|  | Cape Town | 18,3823200 | -33,9227400 |
|  | Cape Town | 18,4225800 | -34,1792100 |
|  | Cape Town | 18,4756600 | -33,5748200 |
|  | Cape Town | 18,4931600 | -34,0359900 |
|  | Cape Town | 18,3682300 | -34,1349300 |
|  | Cape Town | 18,3578600 | -34,0310900 |
|  | Cape Town | 18,4540700 | -33,9228200 |
|  | Cape Town | 18,4799200 | -34,0053300 |
|  | Cape Town | 18,4794800 | -34,0612800 |
|  | Cape Town | 18,4842700 | -33,5844500 |
|  | Cape Town | 18,4885300 | -33,9314300 |
|  | Cape Town | 18,4776100 | -34,0038000 |
|  | Cape Town | 18,4667200 | -34,0966900 |
|  | Cape Town | 18,3823500 | -33,9227700 |
|  | Cape Town | 18,4330700 | -33,9284100 |
|  | Cape Town | 18,4240800 | -33,9240400 |
|  | Cape Town | 18,4551400 | -33,9232600 |
|  | Cape Town | 18,4275600 | -34,1720900 |
|  | Cape Town | 18,4873600 | -33,9520800 |
| Green waste facility | Cape Town | 18,5289100 | -34,0476500 |
|  | Cape Town | 18,5542200 | -34,0253300 |
|  | Cape Town | 18,5362800 | -34,0258200 |
|  | Cape Town | 18,7879700 | -33,9893600 |
|  | Stellenbosch | 18,8048300 | -33,7617500 |
|  | Stellenbosch | 18,7796700 | -33,7661800 |
|  | Cape Town | 18,7013900 | -33,7897000 |
|  | Cape Town | 18,6837000 | -33,8031300 |
|  | Cape Town | 18,7341500 | -33,8776700 |
|  | Cape Town | 18,7425000 | -33,8901400 |
|  | Stellenbosch | 18,7992700 | -33,8409000 |
|  | Stellenbosch | 18,7949900 | -33,7634500 |
|  | Cape Town | 18,8037500 | -34,0541200 |
|  | Cape Town | 18,5693900 | -33,8731800 |
|  | Cape Town | 18,5767400 | -33,8936700 |
|  | Stellenbosch | 18,4268200 | -34,0533500 |
| Landfill | Stellenbosch | 18,8205600 | -33,9421600 |
|  | Cape Town | 18,7064100 | -33,8215000 |
|  | Cape Town | 18,5024900 | -33,7907500 |
|  | Cape Town | 18,7129500 | -33,8784600 |
|  | Cape Town | 18,8473900 | -34,1385800 |
|  | Cape Town | 18,6963600 | -34,0141800 |
|  | Cape Town | 18,5024800 | -34,0911400 |
|  | Cape Town | 18,6532700 | -33,9388600 |
|  | Cape Town | 18,5423200 | -33,7776900 |
|  | Cape Town | 18,6482400 | -34,0520700 |
|  | Cape Town | 18,5431900 | -33,7709500 |
| Mature tree nurseries | Cape Town | 18,8181200 | -34,0248000 |
|  | Cape Town | 18,7559800 | -33,9710800 |
|  | Cape Town | 18,7693000 | -33,9947400 |
|  | Cape Town | 18,7618800 | -33,9922100 |
|  | Cape Town | 18,8097700 | -33,7604200 |
|  | Cape Town | 18,4756000 | -33,9511900 |
|  | Cape Town | 18,7989200 | -34,0588000 |
|  | Cape Town | 18,6491500 | -34,0492700 |
|  | Cape Town | 18,5157100 | -33,9485200 |
